# Multiple-micronutrient supplementation in pregnant adolescents in low- and middle-income countries: a systematic review and a meta-analysis of individual participant data

**DOI:** 10.1093/nutrit/nuab004

**Published:** 2021-04-13

**Authors:** Emily C Keats, Nadia Akseer, Pravheen Thurairajah, Simon Cousens, Zulfiqar A Bhutta, the Global Young Women’s Nutrition Investigators’ Group, Hasmot Ali, Shams El Arifeen, Ulla Ashorn, José Belizan, Robert E Black, Parul Christian, Luz Maria De-Regil, Kathryn Dewey, Michael J Dibley, Wafaie Fawzi, Henrik Friis, Exnevia Gomo, Lieven Huybregts, Renuka Jayatissa, Pernille Kaestel, Subarna K Khatry, Patrick W Kolsteren, Alain B Labrique, Mary McCauley, Brietta M Oaks, Ellen Piwoz, Saijuddin Shaikh, Damayanti D Soekarjo, Christopher R Sudfeld, Willy Urassa, Keith West, Lee Shu-Fune Wu, Noel Zagre, Lingxia Zeng, Zhonghai Zhu

**Affiliations:** 1 Centre for Global Child Health, The Hospital for Sick Children, Toronto, Ontario, Canada; 2 London School of Hygiene & Tropical Medicine, London, United Kingdom; 3 Dalla Lana School of Public Health, University of Toronto, Toronto, Canada; 4 Aga Khan University, Karachi, Pakistan; 5 JiVitA Maternal and Child Nutrition and Health Research Project, Gaibandha, Bangladesh; 6 International Centre for Diarrhoeal Disease Research, Dhaka, Bangladesh; 7 Faculty of Medicine and Health Technology, Tampere University, Tampere, Finland; 8 University of North Carolina at Chapel Hill, Chapel Hill, North Carolina, USA; 9 Johns Hopkins Bloomberg School of Public Health, Baltimore, Maryland, USA; 10 Nutrition International, Ottawa, Ontario, Canada; 11 University of California, Davis, Davis, California, USA; 12 The University of Sydney, Sydney, New South Wales, Australia; 13 Harvard T.H. Chan School of Public Health, Boston, Massachusetts, USA; 14 University of Copenhagen, Copenhagen, Denmark; 15 University of Zimbabwe, Harare, Zimbabwe; 16 International Food Policy Research Institute, Washington, DC, USA; 17 Medical Research Institute, Colombo, Sri Lanka; 18 Nepal Nutrition Intervention Project-Sarlahi, Kathmandu, Nepal; 19 Ghent University, Ghent, Belgium; 20 Centre for Maternal and Newborn Health, Liverpool School of Tropical Medicine, Liverpool, United Kingdom; 21 University of Rhode Island, Kingston, Rhode Island, USA; 22 Bill & Melinda Gates Foundation, Seattle, Washington, USA; 23 Society for Applied Studies, New Delhi, India; 24 Savica Consultancy, Jakarta, Indonesia; 25 Muhimbili University of Health and Allied Sciences, Dar es Salaam, Tanzania; 26 UNICEF Regional Office for West and Central Africa, Dakar, Senegal; 27 School of Public Health, Xi’an Jiaotong University Health Science Centre, Xi’an, Shaanxi, China

**Keywords:** adolescent nutrition, antenatal care, individual participant data meta-analysis, multiple-micronutrient supplementation

## Abstract

**Context:**

Approximately 7.3 million births occur annually among adolescents in low- and middle-income countries. Pregnant adolescents constitute a nutritionally vulnerable group that could benefit from intervention to mitigate the mortality and adverse birth outcomes associated with adolescent pregnancy.

**Objective:**

The aim of this systematic review and meta-analysis was to assess the following: (1) the effect of multiple-micronutrient (MMN) supplementation vs iron and folic acid (IFA) supplementation among adolescents on maternal morbidity, birth outcomes, and mortality outcomes, (2) the effects of MMN supplementation in adolescents compared with the effects in adult women, and (3) the effect modification, if any, of MMN supplementation by baseline and geographic characteristics of adolescents.

**Data Sources:**

MEDLINE and Cochrane databases were searched, along with the reference lists of relevant reviews.

**Study Selection:**

Multiple-micronutrient supplementation trials in pregnancy that were conducted in a low- or middle-income country and had included at least 100 adolescents (10–19 years of age) were eligible for inclusion. Two independent reviewers assessed study eligibility.

**Data Extraction:**

Thirteen randomized controlled trials conducted in Africa and Asia were identified from 1792 reviews and 1578 original trials. Individual-level data was shared by study collaborators and was checked for completeness and extreme values. One- and two-stage individual participant data meta-analyses were conducted using data from randomized controlled trials of MMN supplementation.

**Results:**

A total of 15 283 adolescents and 44 499 adult women with singleton births were included in the individual participant data meta-analyses of MMN supplementation vs IFA supplementation. In adolescents, MMN supplementation reduced low birth weight (1-stage OR = 0.87, 95%CI 0.77–0.97; 2-stage OR = 0.81; 95%CI 0.74–0.88), preterm birth (1-stage OR = 0.88, 95%CI 0.80–0.98; 2-stage OR = 0.86, 95%CI 0.79–0.95), and small-for-gestational-age births (1-stage OR = 0.90, 95%CI 0.81–1.00; 2-stage OR = 0.86, 95%CI 0.79–0.95) when compared with IFA supplementation. The effects of MMN supplementation did not differ between adolescents and older women, although a potentially greater reduction in small-for-gestational-age births was observed among adolescents. Effect modification by baseline characteristics and geographic region was inconclusive.

**Conclusions:**

Multiple-micronutrient supplementation can improve birth outcomes among pregnant adolescents in low- and middle-income countries. Policy related to antenatal care in these settings should prioritize MMN supplementation over the currently recommended IFA supplementation for all pregnant women, especially adolescents.

## INTRODUCTION

Global research and programming priorities in the era of the United Nations (UN) Sustainable Development Goals, established in 2015, have begun to encompass population groups that, up to now, have been left behind. Today, there are 1.2 billion adolescents aged 10 to 19 years who constitute 16% of the world’s population,^1^ and 9 of 10 live in a low- or middle-income country (LMIC).^2^ Within this cohort are 600 million adolescent women (18–19 years) and girls (<18 years)^2^ whose health and well-being is critical for sustainable development.^3^

The nutrition of adolescent girls and adolescent women in LMICs is especially important because of the high burden and severe consequences of malnutrition (encompassing underweight, overweight/obesity, and micronutrient deficiencies) in these settings.^4^ Malnutrition can manifest in several different ways, leading to infections, noncommunicable diseases, disability, or death. Pregnant adolescents are particularly at risk because of the heightened nutritional demands imposed by the fetus coupled with those required for growth during adolescence, with inadequate nutrition having intergenerational consequences for the offspring.^5^ Compared with older women, adolescents are at higher risk of adverse pregnancy outcomes, including maternal morbidities (eg, eclampsia and obstructed labor), stillbirth, preterm birth, and severe neonatal conditions.^6^^,^^7^ In addition, complications of pregnancy are the leading cause of death for girls and women aged 15 to 19 years in LMICs.^8^ Underlying micronutrient deficiencies are prevalent in LMICs, where diet diversity is lacking, and may exacerbate some of these outcomes and negatively impact the growth and development of the infant.^6^^,^^9^ Additionally, multiple pregnancies and short interpregnancy intervals can contribute to poor micronutrient status for mothers of any age.^10^ Given the large number of adolescent girls who will soon enter their reproductive years, the 7.3 million births to adolescents in LMICs per year,^11^ and the importance of good nutrition prenatally and throughout pregnancy, interventions to alleviate this burden of malnutrition will be essential if the Sustainable Development Goals, particularly Sustainable Development Goal 3, are to be achieved.

Currently, the World Health Organization (WHO) recommends daily supplementation with iron and folic acid (IFA) throughout pregnancy,^12^ but recent evidence indicates that a multiple-micronutrient (MMN) supplement provides additional benefits to IFA supplementation and has the advantage of addressing multiple deficiencies simultaneously. A 2019 Cochrane review of 19 randomized controlled trials in LMICs found that supplementation with MMN, when compared with IFA supplementation, can reduce the risk of low birth weight (LBW) and small-for-gestational age (SGA) newborns and may reduce preterm births among women of reproductive age generally.^13^ However, this review did not examine outcomes in adolescents, and the extent to which MMN supplementation benefits pregnant adolescents has received relatively little attention overall, underscoring an important gap in knowledge for evidence-based programming and policy.

Given the current gap, both a one-stage individual participant data (IPD) meta-analysis and a two-stage IPD meta-analysis were conducted to investigate the effect of MMN supplementation in pregnant adolescent girls and women and to identify risk factors relating to the health, survival, and nutritional status of these girls and women and their offspring. Specifically, 3 objectives were addressed: (1) the effect of MMN vs IFA supplementation in pregnant adolescent girls and women; (2) the intervention effect in adolescents compared with that in adult women of reproductive age; and (3) the potential variations in the intervention effect in adolescents by individual-level characteristics and region.

## METHODS

### Mapping of available nutrition-specific intervention data

To identify studies for inclusion in this analysis, a systematic review of systematic reviews and meta-analyses published in 2010 or later was undertaken. Eligible reviews investigated effects of nutrition-specific interventions provided to women of reproductive age in LMICs. This date criterion was applied to the search in order to capture recent data relevant to the research questions. The PICOS (Participants, Intervention, Comparison, Outcome, Study design) criteria were used to translate the research question into a searchable query (Table 1). Searches were conducted in MEDLINE (see Appendix S1 in the Supporting Information online) and the Cochrane Library (adapted MEDLINE search) in September 2016, and the search was updated in March 2020. Studies from relevant systematic reviews (ie, those that met the inclusion criteria) were then assessed by two independent reviewers, and eligibility criteria (see Table S1 in the Supporting Information online) were applied. Reference lists of included reviews and grey literature were also searched. Only individually randomized or cluster-randomized controlled trials were considered for inclusion, and the analysis was restricted to trials that had enrolled at least 100 healthy adolescent girls or women (10–19.9 years). An exception to this was the inclusion of the study by Adu-Afarwuah et al^14^ (N = 71 adolescents) because of the limited availability of lipid-based nutrient supplementation trials. When necessary, trial investigators were contacted for clarification (eg, to determine sample size of adolescents). Risk-of-bias assessment was completed for each trial using the Cochrane risk-of-bias tool, and the results are reported in Appendix S2 in the Supporting Information online. PRISMA (Preferred Reporting Items for Systematic Reviews and Meta-Analyses) guidelines for reporting of systematic reviews were followed; the corresponding checklist can be found in Appendix S3 in the Supporting Information online.

**Table 1:** PICOS criteria for inclusion of studies

Parameter	Description
Population	Women of reproductive age, including adolescents (10–19.9 years of age)
Intervention	Multiple-micronutrient supplementation or lipid-based nutrient supplementation
Comparison	Iron and folic acid supplementation
Outcomes	Birth weight, low birth weight, gestational age, preterm birth, small-for-gestational age birth, stillbirth, perinatal mortality, neonatal mortality, maternal hemoglobin level, maternal anemia
Study design	Individual or cluster-randomized controlled trials

### Formation of the Global Young Women’s Nutrition Investigators’ Group

Investigators of trials that met the inclusion criteria were invited to join the Global Young Women’s Nutrition Investigators’ Group. A complete listing of the Investigators’ Group, which includes 35 members, can be found in Table S2 in the Supporting Information online. For trials that provided supplementation in pregnancy with MMN vs IFA or with lipid-based nutrient supplements (LNS) vs IFA, IPD were requested. Given that LNS contains multiple micronutrients, a prespecified sensitivity analysis was conducted in which outcomes were reexamined when data from MMN and LNS trials were combined (MMN + LNS vs IFA).

### Outcomes and covariates of interest

All outcomes, potential individual-level effect modifiers, and statistical methods were specified a priori. The outcomes were birth weight, LBW (<2500 g), gestational age, preterm birth (<37 weeks of gestation), SGA birth (<10th centile, based on INTERGROWTH-21st standards^15^), stillbirth (fetal death ≥28 weeks of gestation), perinatal mortality (fetal deaths ≥28 weeks of gestation up to day 7 of life), neonatal mortality (death up to day 28 of life), maternal hemoglobin level, and maternal anemia (third trimester hemoglobin <11 g/dL). The covariates were age at enrollment (<20 years vs ≥20 years and <18 years vs 18–19 years), region (Africa vs Asia), parity (primiparous vs multiparous), body mass index (BMI) (underweight [BMI <18.5] vs not underweight [BMI ≥18.5]), gestational age at enrollment (<13 weeks vs ≥13 weeks), maternal height (low stature [<150 cm] vs normal stature [≥150 cm]), maternal education (none vs some), and maternal anemia status at enrollment (anemic [hemoglobin <11 g/dL] vs nonanemic [hemoglobin ≥11 g/dL]).

Because parity could modify the effects of age, the potential age effect (<18 years vs 18–19 years) among primiparous adolescents was also investigated. Investigators provided data sets based on these specifications. Data sets received were assessed to ensure consistency with published reports, and implausible values were excluded (see Table S3 in the Supporting Information online).

### Statistical analysis

While IPD meta-analyses are now considered the gold standard of evidence syntheses, a one-stage approach in which all IPD are analyzed simultaneously in one regression model can have both advantages and disadvantages over a two-stage approach in which aggregate estimates are derived separately for each study and then combined in a fashion similar to a traditional meta-analysis.^16^ There remains some debate surrounding which statistical approach should be adopted in a given situation. Consequently, both a one-stage IPD meta-analysis (primary analysis) and a two-stage IPD meta-analysis were performed, using data from primary trials that were analyzed on an intention-to-treat basis. All multiple births were excluded. Stata software version 15 (StataCorp) was used to conduct all analyses.

Following examination of the age distribution of adolescent girls and women in the trials available, analyses by age within this population focused on the comparison of girls aged less than 18 years with women aged 18 to 19.9 years. Too few girls were aged less than 18 years to allow further subdivision within this group.

### One-stage IPD meta-analysis

The one-stage IPD approach fits a single model to all of the participant-level data simultaneously. For continuous outcomes, linear regression models were used. Results are presented as mean differences (MDs), with 95% confidence limits, between control and intervention groups. For binary outcomes, logistic regression was used. The logistic regression models produced odds ratios (ORs) with 95% confidence limits. With respect to the first and second study objectives, models were fitted that included an interaction term between age and intervention, which allowed age-specific effect estimates to be calculated and a test for effect modification to be performed. For the third objective, the analysis was restricted to women younger than 20 years of age, and again, interactions were included in the models. Potential effect modifiers are listed above (section *Outcomes and covariates of interest*) as covariates of interest. All models included maternal age and study as fixed effects, since both of these variables are predictors of some outcomes and since almost complete data (ie, few missing observations) were available for these variables.

Because of the presence of cluster-randomized trials within the data set, a random-effects model was used to account for between-cluster variation. However, for binary outcomes, fitting models that allowed for differing magnitudes of between-cluster variance in different trials led to convergence problems. Therefore, a constant between-cluster variance was assumed across the cluster-randomized trials. For continuous outcomes, results reported allow for differing between-cluster variation across trials whenever possible (ie, when models converged).

Inclusion of a random effect for treatment was preferred in order to allow for between-study variation in intervention effects, given likely differences in the micronutrient status of women in different trials. However, having between-cluster random effects along with a random effect for treatment caused convergence issues for many models. Where this occurred, fixed effects for treatment were used.

### Two-stage IPD meta-analysis

A two-stage IPD analysis was also performed. Summary parameter estimates were calculated for each study. Estimated parameters included both “main effects” parameters and interaction parameters. Regression models, which controlled for maternal age, were fitted to each study data set using linear regression for continuous outcomes and logistic regression for binary outcomes. For cluster-randomized trials, random-effects models were used to account for the cluster randomization. Study-specific parameter estimates were displayed using forest plots and were combined to obtain summary estimates using the DerSimonian-Laird method.^17^ This method allows for variation of the treatment effect between studies, but when there is no between-study heterogeneity, it will produce the same point estimate and 95%CI as a fixed-effects analysis. Evidence of effect modification was assessed by combining study-specific interaction parameters.

Since IPD from the SUMMIT trial^18^ were not obtained, a second prespecified sensitivity analysis was conducted in which the two-stage IPD results were compared with a two-stage IPD analysis that included published aggregate estimates from the SUMMIT trial.

A third sensitivity analysis was conducted post hoc for selected outcomes in order to test the robustness of findings once the large Bangladeshi trial^19^ was removed, given its oversized contribution to analyses. Two-stage IPD analyses were conducted with and without inclusion of this trial for birth weight and LBW, SGA birth, and maternal anemia.

## RESULTS

**Figure 1:**
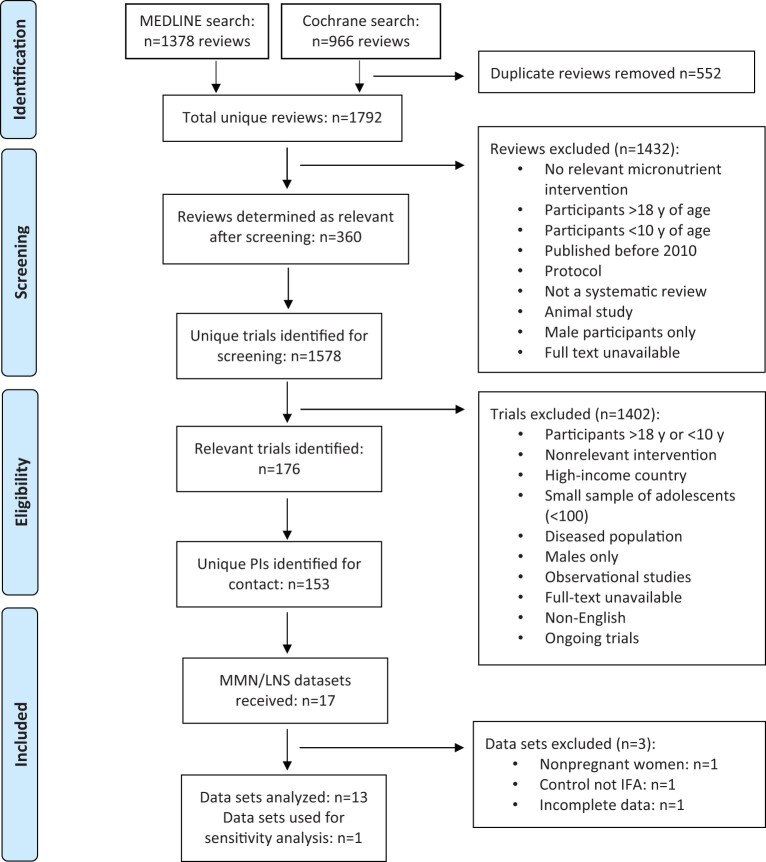
**Flow diagram of the literature search process**. Abbreviations: IFA, iron and folic acid; LNS, lipid-based nutrient supplements; MMN, multiple-micronutrient supplementation; PIs, principal investigators.

From the mapping exercise, 17 relevant MMN/LNS trials were identified (16 MMN trials, 1 LNS trial), of which 13 were included in the one- and two-stage IPD analyses of MMN vs IFA supplementation (Figure 1^14^^,^^19–30^). Three MMN trials were excluded: one was conducted among nonpregnant women^31^; one used a control intervention other than IFA^32^; and, for the SUMMIT trial,^18^ IPD could not be obtained. All other trials provided IPD. Table 2^14^^,^^19–30^ provides a summary of trials included in the main analysis (MMN vs IFA). In total, data for 15 283 healthy pregnant adolescents (10–19 years) and 44 499 healthy older women (≥20 years) were available for analysis. Data were excluded if a woman was HIV positive, had a multiple pregnancy, had a miscarriage or abortion, or was assigned to a trial arm other than IFA or MMN supplementation. One large trial conducted in Bangladesh comprised 48.7% of the total sample (Table 2).^19^ The age distribution of women with data available for analysis by trial can be found in Table S4 in the Supporting Information online. Seven trials provided women with MMN supplements consistent with the UNIMMAP (United Nations International Multiple Micronutrient ) composition (30 mg of iron) proposed by the UN and WHO,^33^ which contains the recommended dietary allowance of 15 vitamins and minerals, while the remaining 6 trials provided women with adapted UNIMMAP supplements that contained an iron concentration of 20 mg, 27 mg, or 60 mg per tablet. All MMN tablets also contained folic acid. Table S5 in the Supporting Information online summarizes the MMN contents and dosage by trial. To better understand the prevalence of the outcomes of interest examined, Table S6 in the Supporting Information reports outcomes by trial from the IFA arm.

**Table 2:** Characteristics of studies included in the individual participant data meta-analysis of multiple-micronutrient supplementation vs iron and folic acid supplementation

Reference	Years of study	Location	Population	No. of women enrolled	No. of data available	No. of adolescent data available	Comments
Adu-Afarwuah et al (2015)^14^	2009–2011	Somanya, and Kpong, Ghana	Pregnant women aged ≥18 y; ≤20 wk of gestation	1320	863	71	440 assigned to LNS were excluded
Ashorn et al (2015)^20^	2011–2013	Mangochi District, Malawi	Pregnant women aged ≥15 y; ≤20 wk of gestation	1391	767	206	462 assigned to LNS were excluded
Bhutta et al (2009)^21^	2002–2004	Bilal Colony, Karachi, Pakistan; Kot Diji, rural Sindh province, Pakistan	Pregnant women <16 wk of gestation; confirmed by ultrasound	2378	2378	209	
Christian et al (2003)^22^	1998–2001	Sarlahi District, Nepal	Pregnant women; pregnancy newly identified by urine test	4926	1677	492	2007 assigned to MMN/IFA; 1645 live births
Fawzi et al (2007)^23^	2001–2004	Dar es Salaam, Tanzania	Pregnant women estimated at 12–27 wk of gestation per date of last menstrual period	8468	8018	1297	
Friis et al (2004)^24^	1996–1997	Harare, Zimbabwe	Pregnant women 22–36 wk of gestation	1669	776	203	526 HIV-positive women excluded; status of 326 pregnancies as singleton/multiple unknown
Kaestel et al (2005)^25^	2001–2002	Bissau, Guinea-Bissau	Pregnant women <37 wk of gestation	2100	1826	389	
Persson et al (2012)^26^	2001–2003	Matlab, Bangladesh	Pregnant women 6–8 wk of gestation; confirmed by urine test and ultrasonography	4436	4387	709	
Osrin et al (2005)^27^	2002–2004	Dhanusha and & Mahottari districts, Nepal	Pregnant women 12–20 wk of gestation with a singleton pregnancy	1200	1200	361	
Roberfroid et al (2008)^28^	2004–2006	Houndé Health District, Burkina Faso	Pregnant women at any gestational age	1426	1331	352	
West et al (2014)^19^	2007–2012	Gaibandha & Rangpur districts, Bangladesh	Pregnant women; pregnancy newly identified by urine test	44 567	29 128	9914	10 126 induced abortions, 4734 miscarriages
Zagre et al (2007)^29^	2004–2006	Maradi, Niger	Pregnant women; pregnancy confirmed by pregnancy test after <12 wk of amenorrhea	3670	3657	771	
Zeng et al (2008)^30^	2002–2006	Shaanxi province, China	Pregnant women ≤28 wk of gestation	5828	3774	309	2017 assigned to FA only excluded
Total				83 379	59 782	15 283	

*Abbreviations*: FA, folic acid; IFA, iron and folic acid supplementation; LNS, lipid-based nutrient supplementation; MMN, multiple-micronutrient supplementation.

**Figure 2:**
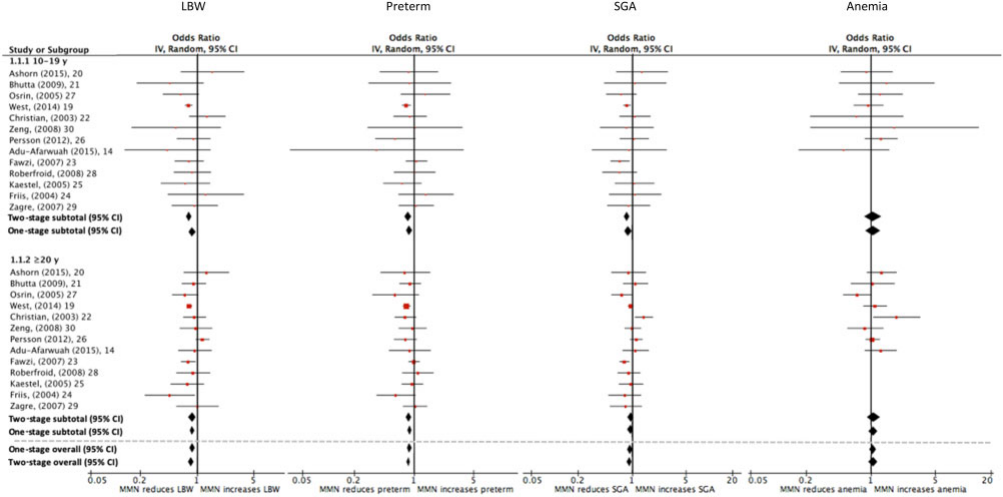
Effect of maternal multiple-micronutrient supplementation containing iron and folic acid compared with iron and folic acid supplementation alone on (a) low birth weight, (b) preterm birth, (c) small-for-gestational-age birth, and (d) maternal anemia when stratified by maternal age (<20 years vs ≥20 years) and using the one-stage and two-stage individual participant data approaches.

**Figure 3:**
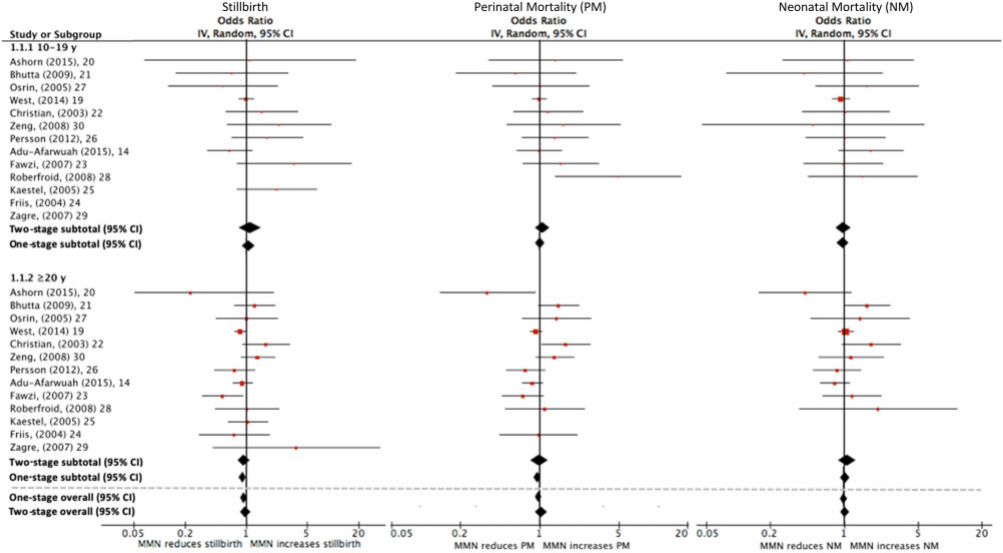
Effect of maternal multiple-micronutrient supplementation containing iron and folic acid compared with iron and folic acid supplementation alone on (a) stillbirth, (b) perinatal mortality, and (c) neonatal mortality when stratified by maternal age (<20 years vs ≥20 years) and using the one-stage and two-stage individual participant data approaches.

Figure 2 ^14^ ^,^ ^19–30^ and Table 3 present the results of the one-stage and two-stage IPD meta-analyses for the following outcomes: LBW, preterm birth, SGA birth, and maternal anemia in the third trimester. Figure 3^14^^,^^19–30^and Table 3 present results for mortality outcomes, including stillbirth, perinatal mortality, and neonatal mortality. When compared with IFA supplementation, MMN supplementation reduced the odds of LBW (one-stage OR = 0.87, 95%CI 0.77–0.97; two-stage OR = 0.81, 95%CI 0.74–0.88) and preterm birth (one-stage OR = 0.88, 95%CI 0.80–0.98; two-stage OR = 0.86, 95%CI 0.79–0.95) among adolescents. Using the two-stage approach, a reduction in SGA births was observed among adolescents who received MMN supplementation (OR = 0.86, 95%CI 0.79–0.95), while the one-stage analysis provided weaker evidence of this (OR = 0.90, 95%CI 0.81–1.00). For the continuous outcomes (Table 4), maternal MMN supplementation provided to adolescents resulted in increased birth weight (one-stage MD = 37 g, 95%CI 15–59; two-stage MD = 49 g, 95%CI 33–65). There was also some indication of a small increase in gestational age (one-stage MD = 0.2 weeks, 95%CI 0.1–0.3; two-stage MD = 0.1 weeks, 95%CI −0.02 to 0.3).

**Table 3:** Effect of maternal multiple-micronutrient supplementation containing iron and folic acid compared with iron and folic acid supplementation alone on low birth weight, preterm birth, SGA birth, stillbirth, neonatal mortality, perinatal mortality, and maternal anemia, when stratified by maternal age (< 20 years vs ≥ 20 years). Results of one-stage and two-stage IPD analysis presented

Outcome		One-stage IPD analysis			Two-stage IPD analysis		
		No. of trials	OR (95%CI)	Test for interaction	No. of trials	OR(95%CI)	Test for interaction
Low birth weight (<2500 g)	All women	13	0.87 (0.80–0.95)^a^		13	0.86 (0.79–0.92)^a^	
	Age <20 y		0.87 (0.77–0.97)^a^	*P* = 0.89		0.81 (0.74–0.88)^a^	*P* = 0.65
	Age ≥20 y		0.87 (0.80–0.95)^a^			0.88 (0.79–0.99)^a^	
Preterm birth (<37 wk)	All women	13	0.89 (0.83–0.95)^a^		13	0.87 (0.83–0.92)^a^	
	Age <20 y		0.88 (0.80–0.98)^a^	*P* = 0.87		0.86 (0.79–0.95)^a^	*P* = 0.67
	Age ≥20 y		0.89 (0.83–0.96)^a^			0.88 (0.82–0.95)^a^	
SGA birth (<10th centile)	All women	13	0.95 (0.87–1.03)		13	0.94 (0.87–1.01)	
	Age <20 y		0.90 (0.81–1.00)^a^	*P* = 0.18		0.86 (0.79–0.94)^a^	*P* = 0.05^a^
	Age ≥20 y		0.96 (0.88–1.04)			0.97 (0.88–1.07)	
Stillbirth (≥28 wk of gestation)	All women	13	0.96 (0.88–1.05)		13	0.98 (0.86–1.13)	
	Age <20 y		1.07 (0.90–1.26)	*P* = 0.15		1.12 (0.82–1.51)	*P* = 0.20
	Age ≥20 y		0.92 (0.83–1.03)			0.95 (0.80–1.12)	
Neonatal mortality (≤28 d)	All women	10	1.02 (0.93–1.13)		10	1.03 (0.93–1.14)	
	Age <20 y		0.98 (0.84–1.16)	*P* = 0.54		0.99 (0.84–1.16)	*P* = 0.83
	Age ≥20 y		1.05 (0.93–1.19)			1.08 (0.88–1.31)	
Perinatal mortality (≥28 wk of gestation up to ≤7 d	All women	11	0.99 (0.92–1.06)		11	1.04 (0.92–1.18)	
	Age <20 y		1.04 (0.92–1.18)	*P* = 0.32		1.07 (0.91–1.25)	*P* = 0.28
	Age ≥20 y		0.96 (0.88–1.05)			1.00 (0.84–1.20)	
Maternal anemia (Hb in 3rd trimester <110 g/L)	All women	8	1.05 (0.95–1.18)		8	1.06 (0.95–1.19)	
	Age <20 y		1.07 (0.86–1.33)	*P* = 0.94		1.06 (0.86–1.31)	*P* = 0.85
	Age ≥20 y		1.06 (0.94–1.18)			1.08 (0.91–1.27)	

*Abbreviations:* Hb, hemoglobin; IPD, individual participant data; OR, odds ratio; SGA, small-for-gestational age.

a Statistically significant estimate.

**Table 4:** Effect of maternal multiple-micronutrient supplementation containing iron and folic acid compared with iron and folic acid alone on birth weight (grams), gestational age (weeks), and maternal hemoglobin level (g/dL), when stratified by maternal age (< 20 years vs ≥ 20 years). Results of one-stage and two-stage IPD analysis presented

Outcome		One-stage IPD analysis			Two-stage IPD analysis		
		No. of trials	MD (95%CI)	Test for interaction	No. of trials	MD (95%CI)	Test for interaction
Birth weight (grams)	All women	13	+38 (22–55)^a^		13	+40 (27–54)^a^	
	Age < 20 y		+37 (15–59)^a^	*P* = 0.89		+49 (33–65)^a^	*P* = 0.87
	Age ≥ 20 y		+39 (22–55)^a^			+36 (16–56)^a^	
Gestational age (weeks)	All women	13	+0.2 (0.1–0.3)^a^		13	+0.2 (0.1–0.3)^a^	
	Age < 20 y		+0.2 (0.1–0.3)^a^	*P* = 0.82		+0.1 (−0.02 to 0.3)	*P* = 0.46
	Age ≥20 y		+0.2 (0.1–0.3)^a^			+0.2 (0.1–0.3)^a^	
Maternal Hb level (g/dL)	All women	9	−0.03 (−0.09 to 0.02)		9	−0.05 (−0.12 to 0.02)	
	Age < 20 y		−0.09 (−0.21 to 0.04)	*P* = 0.36		−0.1 (−0.22 to 0.02)	*P* = 0.67
	Age ≥ 20 y		−0.02 (−0.09 to 0.04)			−0.04 (−0.12 to 0.05)	

*Abbreviations*: Hb, hemoglobin; IPD, individual participant data; MD, mean difference.

a Statistically significant estimate.

To assess whether the intervention effect differs between adolescents and adult women of reproductive age, the test for effect modification by age (<20 years vs ≥20 years) for each dichotomous outcome (Table 3) and continuous outcome (Table 4) was reported. Age did not appear to modify the effect of MMN supplementation for any outcome examined (*P* for interaction >0.1 for all outcomes), with the possible exception of SGA births. There appeared to be a greater positive effect of MMN supplementation on SGA births for adolescents (two-stage OR = 0.86, 95%CI 0.79–0.94) than for older women of reproductive age (OR = 0.97, 95%CI 0.88–1.07; *P* for interaction = 0.05). Results from the one-stage analysis revealed a similar pattern, but with weaker evidence of effect modification (*P* for interaction = 0.18).

To examine whether the intervention effect in adolescents varies by individual-level characteristics, additional factors that could potentially modify the effect of MMN supplementation in adolescent women and girls were examined (Table 5). There was some evidence to suggest that maternal BMI and gestational age at enrollment modified the effect of MMN supplementation on stillbirths among adolescents. The odds of stillbirth were increased among normal-weight adolescents (OR = 1.26, 95%CI 1.02–1.55) but tended to be reduced among underweight adolescents (OR = 0.79, 95%CI 0.58–1.07; *P* for interaction = 0.01). Additionally, the odds of stillbirth were increased among those who began supplementation before 13 weeks of gestation (OR = 1.31, 95%CI 1.06–1.63) but reduced among those who started supplementation later in pregnancy (OR = 0.75, 95%CI 0.56–0.99; *P* for interaction = 0.002). However, given the large number of statistical tests performed for this analysis, *P* values should be interpreted in that light (ie, only a very small *P* value among 180 *P* values would be convincing of an effect).^34^

**Table 5:** Effect of maternal multiple-micronutrient supplementation on outcomes in adolescent women, stratified by potential effect modifiers (maternal age, region, parity, maternal BMI, gestational age at enrollment, maternal height, maternal education, and maternal hemoglobin level)

Outcome	Characteristic	Level	OR (95%CI)	Test for interaction
Low birth weight (RE)	Maternal age	<18 y	0.77 (0.68–0.88)	*P* = 0.42
		18–19 y	0.83 (0.74–0.94)	
	Maternal age (among primiparous women only)	<18 y	0.75 (0.64–0.86)	*P* = 0.38
		18–19 y	0.81 (0.71–0.94)	
	Region	Africa	0.87 (0.68–1.10)	*P* = 0.55
		Asia	0.81 (0.66–0.99)	
	Parity	Primiparous	0.78 (0.70–0.87)	*P* = 0.73
		Multiparous	0.81 (0.66–1.01)	
	Maternal BMI	<18.5	0.79 (0.67–0.92)	*P* = 0.75
		≥18.5	0.81 (0.72–0.91)	
	GA at enrollment	<13 wk	0.81 (0.72–0.90)	*P* = 0.95
		≥13 wk	0.81 (0.70–0.94)	
	Maternal height	<150 cm	0.83 (0.73–0.94)	*P* = 0.52
		≥150 cm	0.78 (0.69–0.89)	
	Maternal education	None	0.80 (0.66–0.98)	*P* = 0.98
		Some	0.81 (0.73–0.90)	
	Maternal Hb	<11 g/dL	0.79 (0.59–1.06)	*P* = 0.86
		≥11 g/dL	0.82 (0.62–1.10)	
Preterm birth (RE)	Maternal age	<18 y	0.80 (0.69–0.92)	*P* = 0.23
		18–19 y	0.89 (0.79–1.01)	
	Maternal age (among primiparous women only)	<18 y	0.80 (0.70–0.93)	*P* = 0.17
		18–19 y	0.92 (0.80–1.05)	
	Region	Africa	1.00 (0.83–1.21)	*P* = 0.10
		Asia	0.82 (0.74–0.91)	
	Parity	Primiparous	0.84 (0.76–0.94)	*P* = 0.63
		Multiparous	0.90 (0.72–1.12)	
	Maternal BMI	<18.5	0.82 (0.69–0.97)	*P* = 0.51
		≥18.5	0.87 (0.78–0.97)	
	GA at enrollment	<13 wk	0.84 (0.74–0.95)	*P* = 0.51
		≥13 wk	0.89 (0.77–1.03)	
	Maternal height	<150 cm	0.80 (0.69–0.92)	*P* = 0.25
		≥150 cm	0.88 (0.78–1.00)	
	Maternal education	None	0.98 (0.81–1.19)	*P* = 0.07
		Some	0.80 (0.72–0.90)	
	Maternal Hb	<11 g/dL	1.04 (0.82–1.33)	*P* = 0.09
		≥11 g/dL	0.76 (0.58–1.01)	
SGA birth (RE)	Maternal age	<18 y	0.78 (0.69–0.89)	*P* = 0.05
		18–19 y	0.93 (0.83–1.03)	
	Maternal age (among primiparous women only)	<18 y	0.75 (0.65–0.87)	*P* = 0.16
		18–19 y	0.86 (0.76–0.98)	
	Region	Africa	0.81 (0.67–0.99)	*P* = 0.52
		Asia	0.87 (0.80–0.96)	
	Parity	Primiparous	0.81 (0.74–0.90)	*P* = 0.09
		Multiparous	0.98 (0.80–1.19)	
	Maternal BMI	<18.5	0.95 (0.82–1.11)	*P* = 0.15
		≥18.5	0.83 (0.75–0.92)	
	GA at enrollment	<13 wk	0.87 (0.78–0.97)	*P* = 0.95
		≥13 wk	0.87 (0.76–0.99)	
	Maternal height	<150 cm	0.93 (0.82–1.05)	*P* = 0.13
		≥150 cm	0.82 (0.73–0.92)	
	Maternal education	None	0.85 (0.71–1.03)	*P* = 0.96
		Some	0.86 (0.78–0.94)	
	Maternal Hb	<11 g/dL	0.81 (0.63–1.03)	*P* = 0.23
		≥11 g/dL	0.99 (0.78–1.25)	
Stillbirth (FE)	Maternal age	<18 y	1.14 (0.88–1.47)	*P* = 0.54
		18–19 y	1.03 (0.82–1.28)	
	Maternal age (among primiparous women only)	<18 y	1.13 (0.86–1.48)	*P* = 0.77
		18–19 y	1.07 (0.83–1.38)	
	Region	Africa	1.41 (0.55–3.62)	*P* = 0.90
		Asia	1.03 (0.86–1.24)	
	Parity	Primiparous	1.09 (0.91–1.31)	*P* = 0.11
		Multiparous	0.72 (0.45–1.17)	
	Maternal BMI	<18.5	0.79 (0.58–1.07)	*P* = 0.01^a^
		≥18.5	1.26 (1.02–1.55)	
	GA at enrollment	<13 wk	1.31 (1.06–1.63)	*P* = 0.002^a^
		≥13 wk	0.75 (0.56–0.99)	
	Maternal height	<150 cm	1.03 (0.82–1.31)	*P* = 0.49
		≥150 cm	1.17 (0.91–1.50)	
	Maternal education	None	1.07 (0.75–1.53)	*P* = 0.96
		Some	1.06 (0.87–1.29)	
	Maternal Hb	<11 g/dL	0.81 (0.48–1.36)	*P* = 0.36
		≥11 g/dL	1.15 (0.66–1.98)	
Neonatal mortality (FE)	Maternal age	<18 y	0.95 (0.75–1.20)	*P* = 0.69
		18–19 y	1.01 (0.82–1.25)	
	Maternal age (among primiparous women only)	<18 y	0.96 (0.75–1.23)	*P* = 0.88
		18–19 y	0.99 (0.78–1.25)	
	Region	Africa	1.38 (0.86–2.19)	*P* = 0.17
		Asia	0.94 (0.79–1.12)	
	Parity	Primiparous	0.98 (0.82–1.16)	*P* = 0.99
		Multiparous	0.97 (0.60–1.59)	
	Maternal BMI	<18.5	0.97 (0.73–1.27)	*P* = 0.92
		≥18.5	0.99 (0.80–1.21)	
	GA at enrollment	<13 wk	0.99 (0.80–1.21)	*P* = 0.97
		≥13 wk	0.98 (0.76–1.26)	
	Maternal height	<150 cm	1.01 (0.82–1.26)	*P* = 0.59
		≥150 cm	0.93 (0.73–1.18)	
	Maternal education	None	1.12 (0.79–1.61)	*P* = 0.43
		Some	0.95 (0.79–1.14)	
	Maternal Hb	<11 g/dL	1.39 (0.82–2.34)	*P* = 0.82
		≥11 g/dL	1.28 (0.76–2.14)	
Perinatal mortality (FE)	Maternal age	<18 y	1.00 (0.83–1.20)	*P* = 0.59
		18–19 y	1.07 (0.91–1.26)	
	Maternal age (among primiparous women only)	<18 y	1.01 (0.83–1.23)	*P* = 0.64
		18–19 y	1.08 (0.90–1.30)	
	Region	Africa	1.87 (1.05–3.34)	*P* = 0.08
		Asia	1.01 (0.89–1.16)	
	Parity	Primiparous	1.05 (0.92–1.20)	*P* = 0.24
		Multiparous	0.83 (0.58–1.19)	
	Maternal BMI	<18.5	0.84 (0.68–1.05)	*P* = 0.02
		≥18.5	1.16 (1.00–1.36)	
	GA at enrollment	<13 wk	1.15 (0.98–1.35)	*P* = 0.03
		≥13 wk	0.87 (0.71–1.06)	
	Maternal height	<150 cm	1.05 (0.88–1.24)	*P* = 0.99
		≥150 cm	1.04 (0.87–1.26)	
	Maternal education	None	1.11 (0.84–1.47)	*P* = 0.59
		Some	1.02 (0.89–1.17)	
	Maternal Hb	<11 g/dL	1.11 (0.75–1.63)	*P* = 0.86
		≥11 g/dL	1.16 (0.77–1.76)	
Maternal anemia (RE)	Maternal age	<18 y	0.82 (0.58–1.18)	*P* = 0.08
		18–19 y	1.21 (0.93–1.59)	
	Maternal age (among primiparous women only)	<18 y	0.77 (0.52–1.14)	*P* = 0.16
		18–19 y	1.09 (0.80–1.49)	
	Region	Africa	0.78 (0.45–1.37)	*P* = 0.29
		Asia	1.06 (0.89–1.40)	
	Parity	Primiparous	0.98 (0.76–1.25)	*P* = 0.09
		Multiparous	1.69 (0.94–3.05)	
	Maternal BMI	<18.5	1.16 (0.69–1.96)	*P* = 0.65
		≥18.5	1.04 (0.71–1.54)	
	GA at enrollment	<13 wk	1.13 (0.85–1.50)	*P* = 0.55
		≥13 wk	0.99 (0.72–1.37)	
	Maternal height	<150 cm	1.09 (0.80–1.48)	*P* = 0.90
		≥150 cm	1.06 (0.80–1.41)	
	Maternal education	None	1.05 (0.65–1.68)	*P* = 0.86
		Some	1.00 (0.79–1.28)	
	Maternal Hb	<11 g/dL	1.01 (0.66–1.54)	*P* = 0.74
		≥11 g/dL	0.92 (0.66–1.28)	
Birth weight (grams) (RE)	Maternal age	<18 y	MD=57 (32–83)	*P* = 0.54
		18–19 y	MD=47 (27–68)	
	Maternal age (among primiparous women only)	<18 y	MD=66 (39–94)	*P* = 0.40
		18–19 y	MD=51 (27–75)	
	Region	Africa	MD=48.1 (7.7–88.5)	*P* = 0.95
		Asia	MD=49.2 (31.5–66.8)	
	Parity	Primiparous	MD=57 (39–76)	*P* = 0.35
		Multiparous	MD=37 (−1 to 76)	
	Maternal BMI	<18.5	MD=50 (21–79)	*P* = 0.93
		≥18.5	MD=51 (32–71)	
	GA at enrollment	<13 wk	MD=54 (33–76)	*P* = 0.69
		≥13 wk	MD=48 (23–72)	
	Maternal height	<150 cm	MD=43 (19–68)	*P* = 0.46
		≥150 cm	MD=56 (34–77)	
	Maternal education	None	MD=56 (18–93)	*P* = 0.81
		Some	MD=50 (32–69)	
	Maternal Hb	<11 g/dL	MD=46 (−1 to 93)	*P* = 0.63
		≥11 g/dL	MD=63 (14–111)	
Gestational age (weeks) (RE)	Maternal age	<18 y	MD=0.3 (0.1–0.4)	*P* = 0.47
		18–19 y	MD=0.2 (0.1–0.3)	
	Maternal age (among primiparous women only)	<18 y	MD=0.3 (0.1–0.5)	*P* = 0.11
		18–19 y	MD=0.1 (−0.03 to 0.3)	
	Region	Africa	MD=0.15 (−0.14 to 0.43)	*P* = 1.00
		Asia	MD=0.14 (−0.07 to 0.34)	
	Parity	Primiparous	MD=0.2 (0.1–0.3)	*P* = 0.16
		Multiparous	MD=0.4 (0.1–0.7)	
	Maternal BMI	<18.5	MD=0.3 (0.1–0.5)	*P* = 0.60
		≥18.5	MD=0.2 (0.1–0.4)	
	GA at enrollment	<13 wk	MD=0.3 (0.2–0.4)	*P* = 0.15
		≥13 wk	MD=0.1 (−0.01 to 0.3)	
	Maternal height	<150 cm	MD=0.3 (0.2–0.5)	*P* = 0.13
		≥150 cm	MD=0.2 (0.03–0.3)	
	Maternal education	None	MD=0.1 (−0.1 to 0.4)	*P* = 0.42
		Some	MD=0.3 (0.1–0.4)	
	Maternal Hb	<11 g/dL	MD=−0.1 (−0.3 to 0.2)	*P* = 0.06
		≥11 g/dL	MD=0.3 (0.04–0.6)	
Maternal Hb (g/dL) (RE)	Maternal age	<18 y	MD=0.01 (−0.21 to 0.23)	*P* = 0.22
		18–19 y	MD=−0.16 (−0.32 to 0.01)	
	Maternal age (among primiparous women only)	<18 y	MD=0.04 (−0.20 to 0.28)	*P* = 0.35
		18–19 y	MD=−0.10 (−0.29 to 0.08)	
	Region	Africa	MD=−0.25 (−1.09 to 0.59)	*P* = 0.85
		Asia	MD=−0.10 (−0.22 to 0.03)	
	Parity	Primiparous	MD=−0.05 (−0.19 to 0.10)	*P* = 0.30
		Multiparous	MD=−0.25 (−0.62 to 0.11)	
	Maternal BMI	<18.5	MD=−0.12 (−0.37 to 0.14)	*P* = 0.89
		≥18.5	MD=−0.10 (−0.25 to 0.06)	
	GA at enrollment	<13 wk	MD=−0.09 (−0.27 to 0.09)	*P* = 0.77
		≥13 wk	MD=−0.13 (−0.32 to 0.07)	
	Maternal height	<150 cm	MD=0.05 (−0.14 to 0.24)	*P* = 0.03
		≥150 cm	MD=−0.23 (−0.41 to −0.05)	
	Maternal education	None	MD=0.03 (−0.28 to 0.35)	*P* = 0.42
		Some	MD=−0.11 (−0.26 to 0.04)	
	Maternal Hb	<11 g/dL	MD=−0.17 (−0.44 to 0.10)	*P* = 0.25
		≥11 g/dL	MD=0.02 (−0.17 to 0.21)	

*Abbreviations:* BMI, body mass index; FE, fixed effects; GA, gestational age; Hb, hemoglobin; MD, mean difference; RE, random effects; SGA, small for gestational age.

a Statistically significant (*P* < 0.05).

As a sensitivity analysis, a two-stage IPD analysis that included published aggregate estimates from the SUMMIT trial^18^ (for which IPD could not be obtained) (see Table S7 in the Supporting Information online) was conducted and compared with a two-stage IPD without the SUMMIT data (Table 3). For outcomes for which SUMMIT data were available (LBW, preterm birth, SGA birth, stillbirth, and neonatal mortality), there was no important statistical differences between the two analyses.

As a second sensitivity analysis, outcomes were examined when data from MMN and LNS trials were combined (see Table S8 in the Supporting Information online) using the one-stage approach. This allowed for the inclusion of one additional trial^35^ along with LNS arms from two MMN trials that had not been previously included.^14^^,^^20^ This strengthened somewhat the evidence that MMN/LNS reduced the risk of SGA birth (OR = 0.88, 95%CI 0.79–0.97) among adolescents.

As a third sensitivity analysis, the outcomes of birth weight and LBW (see Tables S9 and S10 in the Supporting Information online), SGA births (see Table S11 in the Supporting Information online), and maternal anemia (see Table S12 in the Supporting Information online) were examined after removal of data from the large Bangladeshi trial.^19^ This produced slightly weaker evidence that MMN supplementation reduced the risk of LBW among adolescents (OR = 0.86, 95%CI 0.72–1.03), though a similar effect was noted for older women of reproductive age (OR = 0.90, 95%CI 0.80–1.03; *P* for interaction = 0.65). Evidence of an effect on SGA birth among adolescents (OR = 0.86, 95%CI 0.74–1.00) became slightly more uncertain, though the point estimate remained the same, and there was weaker evidence of effect modification by age (OR ≥20 years = 0.97, 95%CI 0.86–1.11; *P* for interaction = 0.36). However, given the removal of 47%, 47%, and 19% of the total sample for birth weight/LBW, SGA birth, and maternal anemia, respectively, it was anticipated that estimates would become less precise (ie, 95%CIs became wider for each outcome). No other major differences were noted.

## DISCUSSION

Multiple-micronutrient supplementation in pregnant adolescents reduced the odds of LBW, preterm births, and SGA births. Translating this benefit to a risk ratio, there was an 8% decline in infants born with LBW and an 8% decline in preterm births among adolescents who received MMN supplements compared with current standard of care (IFA supplements). There was suggestive evidence that MMN benefits adolescents (10–19 years) more than older women of reproductive age (≥20 years) with regard to SGA births, but not other outcomes. In addition, there was some weak evidence to suggest that individual-level factors, such as timing of supplementation initiation and maternal nutritional status at baseline, modified the effect of MMN supplementation on infant survival. Results from the one-stage and two-stage IPD analyses were generally similar and led to similar conclusions in terms of impact. For SGA births among adolescents, a slightly greater effect was shown with the two-stage IPD analysis, which likely reflects methodological differences, including model assumptions, between the two IPD analyses.

Several limitations to this work should be noted. First, one-stage analyses that include cluster-randomized trials can lead to computational difficulties, especially with binary outcomes and when trying to include random treatment effects. For computations to be tractable, the between-cluster variance was assumed to be the same for all cluster-randomized trials. For this reason, both a one-stage IPD meta-analysis and a two-stage IPD analysis were conducted. Second, the current analysis is based on a very large number of statistical tests (ie, 180 tests were conducted for effect modification; Table 5); in this situation, some small *P* values should be expected by chance. Specifically, 9 of these tests would be significant at *P* < 0.05, by chance. Considering that fewer than 9 *P*-for-interaction values less than 0.05 were reported, it is likely that all of them are due to chance or can be considered false-positive results within the analyses. Third, because adolescent mothers were not specifically recruited for these trials, the sample of younger adolescents (10–14 years) was too small to perform meaningful effect modification within this group or to compare younger adolescents with older adolescents. Younger adolescents are biologically different from older adolescents, and some studies have suggested that pregnancy risks and birth complications are increased for this group.^36^^,^^37^ Therefore, the effects of nutrition interventions, including MMN supplementation, could potentially be different within this subset of mothers. More research is required to determine this, but it would be practically challenging because of the need for a large population of pregnant girls aged 10 to 14 years.

Data on infant sex were not requested, and therefore it could not be determined whether there was an enhanced survival effect for female infants born to adolescent mothers who were supplemented with MMN, as has been shown for pregnant women of all ages in other meta-analyses.^19^^,^^38^^,^^39^ It has been suggested that this may be mediated through a longer gestational period and improved intrauterine growth for female, but not male, infants.^19^^,^^38^^,^^39^ Data from the SUMMIT trial,^18^ a large MMN supplementation trial (31 290 women) conducted in Indonesia, are also missing. While the sensitivity analysis revealed no major differences in effect when SUMMIT data were included or were not included in a traditional meta-analysis, incorporating the IPD within the one-stage approach would have increased completeness and reduced dependence on the West et al^19^ study (JiVitA-3 trial), which contributed almost half of the sample to the data set and thus was weighted heavily in the analyses. Lastly, the important differences in population characteristics, cointerventions (eg, malaria prophylaxis), and MMN content and micronutrient dosage within the trial data and their potential effects on the findings should be acknowledged. However, the aim of this meta-analysis was to determine the impact of MMN supplementation in adolescent mothers across LMICs, and therefore it is appropriate as a first-cut technique to pool data that will maximize sample size and provide a broad indication of the impact of the intervention.

Compared with other recent evidence syntheses on this topic, the present analysis shows some similarities and some differences, which are likely the result of the differential inclusion and exclusion of studies. In their analysis of 17 trials (112 953 women) Smith et al^38^ used IPD in a two-stage analytic approach to examine potential individual-level modifiers of the effect of MMN supplementation. Maternal age (<20 years, ≥20 years) was one of the potential effect modifiers. These authors also found no important differences by maternal age for any of the outcomes examined and noted a greater benefit of supplementation for women with nutritional deficiency (indicated by low BMI or anemia status). This analysis demonstrated a trend toward a reduction in stillbirth and perinatal mortality outcomes for underweight vs normal-weight adolescent women.

The 2019 Cochrane update (N = 19 trials; 141 447 women)^13^ revealed a greater reduction in preterm births for underweight women, a greater reduction in SGA births for normal-weight and normal-stature (≥154.9 cm) women, and greater reductions in SGA births and perinatal mortality when supplementation was initiated after 20 weeks of gestation.^13^ Some benefits were observed with later initiation of MMN supplementation (13 weeks of gestation or later compared with <13 weeks) among adolescent girls and women in this analysis.

Within the analysis by Smith et al,^38^ the 2019 Cochrane update,^13^ and the current analysis, there was no indication of increased risk of stillbirth, neonatal mortality, perinatal mortality, or infant/child mortality with MMN supplementation in pregnancy overall. However, the tests for effect modification provide some evidence that stillbirths and perinatal deaths (largely driven by stillbirths) could be increased among adolescents with BMI ≥18.5 who receive MMN supplementation. Similar effects for adolescent mothers who began supplementation prior to 13 weeks of gestation were noted, a finding that could be biologically plausible based on the notion that longer supplementation periods may lead to larger infants, which could, in turn, increase the risk of birth complications such as asphyxia and several forms of dysfunctional labor.^40^^,^^41^ However, given this line of biological reasoning, one might then assume that the impact among short adolescent mothers would be comparable or even greater, yet stature under 150 cm was not found to increase the risk of stillbirths with MMN supplementation. In addition, women with normal BMI vs women with low BMI had similar outcomes for LBW, preterm birth, SGA birth, birth weight (grams), and gestational age (weeks). There is also the potential for imbalanced confounding (when randomized assignment has not been preserved) and bias when examining effect modification, as it was done by conducting a stratified analysis by risk categories within the smaller subsample of adolescents. Given the inconsistency of findings for stillbirths compared with other outcomes and the multiplicity of statistical tests performed, these results are probably due to chance and are likely spurious.

Until recently, there has been limited evidence to support policy and programming initiatives for adolescent mothers in LMICs, despite WHO guidelines on antenatal care for a positive pregnancy experience encompassing all pregnant women and adolescent girls.^12^ For example, no antenatal MMN supplementation trials focused solely on the adolescent population have been conducted. An ongoing trial in Pakistan could help to clarify certain remaining questions. In this large-scale trial, adolescent and young women (15–24 years) are provided with a cointervention of life-skills education and MMN supplements during preconception (twice weekly), pregnancy (daily), and postpartum (daily to 6 months) or standard of care (daily IFA and nonregulated, community-based health sessions) to examine both maternal and infant outcomes.^42^ Given the potential nutritional vulnerabilities of pregnant adolescents, coupled with the high number of adolescent pregnancies in LMICs, there is a strong case for targeting pregnant adolescents deliberately to ensure they receive nutritional supplementation and quality antenatal and obstetric services. The concurrent use of targeted strategies for adolescents, such as the use of promotional platforms (eg, social media, mHealth)^6^, could work beneficially to improve the awareness and uptake of services and, eventually, to improve health outcomes among adolescent mothers and their infants.

## CONCLUSION

The analyses presented here suggest that the impact of MMN supplementation does not differ substantially between adolescents and adult women. However, younger adolescent girls may have different risks related to nutrient requirements, nutrient intakes, and health and birth outcomes when compared with older adolescent girls and women. While the policy approach should aim to prevent adolescent pregnancy, further targeted research on the mechanisms of benefit among the youngest mothers would be useful when adolescent pregnancy does occur. Given these findings, which are in line with recent efforts to examine the usefulness of MMN vs IFA supplementation in pregnancy,^43^ it is recommend that the global nutrition community support policy and programming efforts to implement and expand the use of MMN supplementation, in preference to IFA supplementation alone, for pregnant women, including adolescents in low- and middle-income settings. The cost per MMN tablet is slightly higher than that of IFA^44^; however, given the existing channels for IFA distribution, the additional costs of commodity change would be minimal while the benefits on birth outcomes would be potentially greater, especially when implementation is achieved at scale.^44^ While current evidence from the subset of MMN trials that report on long-term health and neurodevelopmental outcomes does not suggest benefits,^45^ future studies to capture such outcomes are warranted. The UN Sustainable Development Goals will be realized only when the nutritional health and well-being of this critical cohort of adolescents is optimized.

## Supplementary Material

nuab004_Supplementary_Data
