# Supplementary material for: Multiple-micronutrient supplementation in pregnant adolescents in low- and middle-income countries: a systematic review and a meta-analysis of individual participant data
Source: Nutr Rev. 2021 Apr 13;80(2):141–56. doi: 10.1093/nutrit/nuab004 (PMC8754251; doi:10.1093/nutrit/nuab004)
Supplement: nuab004_Supplementary_Data [file nuab004_supplementary_data.docx]

**Appendix S1: Additional Tables**

**Table S1:** Eligibility criteria for studies.

| **INCLUSION CRITERIA** |
| --- |
| Randomized or cluster-randomized controlled trial |
| Conducted in a low or middle-income country |
| At least 100 adolescent (10-19.9 years) women included in study sample^1^ |
| Examining multiple micronutrient or lipid-nutrient supplementation compared to iron-folic acid supplementation |
| **EXCLUSION CRITERIA** |
| Ongoing trial |
| Conducted in an unhealthy population (e.g., with chronic or infectious disease) |
| Non-English language |

^1^An exception to this was the inclusion of one trial (Adu-Afarwuah 2015) (N=71 adolescents) because of the limited availability of lipid-nutrient supplementation trials.

**Table S2:** Members of Investigators Group and Expert Advisory Committee

| **INVESTIGATOR** | **INSTITUTION** | **INVESTIGATOR** | **INSTITUTION** |
| --- | --- | --- | --- |
| Faruk Ahmed | Griffiths University, Australia | Mary McCauley | Liverpool School of Tropical Medicine, UK |
| Ulla Ashorn | University of Tampere, Finland | Bright Nwaru | University of Edinburgh, Scotland |
| Jose Belizan | UNC Chapel Hill, USA | Brietta Oaks | University of Rhode Island, USA |
| Zulfiqar Bhutta | Centre for Global Child Health, Canada and Aga Khan University, Pakistan | Saskia Osendarp | Wageningen University, Netherlands |
| Robert Black | JHSPH, USA | David Osrin | University College London, UK |
| Parul Christian | JHSPH, USA | Luz Maria de Regil | Nutrition International, Canada |
| Caroline Crowther | University of Adelaide, Australia | David Ross | WHO, Switzerland |
| Kay Dewey | UC Davis, USA | Alice Rumbold | University of Adelaide, Australia |
| Michael Dibley | University of Sydney, Australia | Damayanti Soekarjo | Savica, Indonesia |
| Shams El Arifeen | ICDDRB, Bangladesh | Nynke Van den Broek | Liverpool School of Tropical Medicine, UK |
| Wafaie Fawzi | Harvard University, USA | Anuraj Shankar | Harvard University, USA |
| Exnevia Gomo | University of Zimbabwe, Zimbabwe | Sreenivas Vishnubhatla | All India Institute of Medical Sciences, India |
| Ravinder Goswami | All India Institute of Medical Sciences, India | Keith West | JHSPH, USA |
| Lieven Huybregts | International Food Policy Research Institute, USA | Lee Wu | JHSPH, USA |
| Renuka Jayatissa | Ministry of Health, Sri Lanka | Noel Zagre | UNICEF, Western and Central Regional Office |
| Pernille Kaestel | University of Copenhagen, Denmark | Lingxia Zeng | Xi’an Jiaotong University, China |
| Patrick Kolsteren | Ghent University, Belgium | Stanley Zlotkin | University of Toronto, Canada |
| Tjalling Leenstra | National Institute for Public Health and the Environment, Netherlands |  |  |

**Table S3:** Criteria for exclusion of implausible values.

| **Variable** | **Cut-offs** |
| --- | --- |
| Maternal height | ≤100 cm |
| Maternal weight | <18 kg |
| Gestational age at birth | <22 weeks |
|  | ≥45 weeks |
| Birthweight^1^ | <340 grams |
|  | >5180 grams |
| Birth length^1^ | <24 cm |
|  | >56.7 cm |
| Head circumference^1^ | <17.4 cm |
|  | >39 cm |
| ANC visits | >20 visits |

^1^Based on Intergrowth-21^st^ values (>3 SD below average at 24 weeks gestation or >3 SD above average at 42 weeks gestation)

**Table S4:** Age distribution of women available for analysis by trial.

| **Study** | **<15** | **15-17** | **18-19** | **20-24** | **25-29** | **30+** | **Total** |
| --- | --- | --- | --- | --- | --- | --- | --- |
|  |  |  |  |  |  |  |  |
| Adu-Afarwuah | 0 | 0 | 71 | 270 | 284 | 238 | 863 |
| Ashorn | 1 | 86 | 119 | 231 | 190 | 140 | 767 |
| Bhutta | 4 | 60 | 145 | 772 | 807 | 590 | 2378 |
| Christian | 20 | 232 | 240 | 592 | 345 | 248 | 1677 |
| Fawzi | 0 | 10 | 1287 | 3225 | 2117 | 1379 | 8018 |
| Friis | 3 | 75 | 125 | 310 | 150 | 113 | 776 |
| Kaestel | 3 | 131 | 255 | 648 | 466 | 323 | 1826 |
| MINIMat | 14 | 249 | 446 | 1306 | 1195 | 1177 | 4387 |
| Osrin | 0 | 102 | 259 | 584 | 201 | 54 | 1200 |
| Roberfroid | 1 | 154 | 197 | 414 | 279 | 286 | 1331 |
| West | 397 | 4334 | 5183 | 9443 | 6059 | 3712 | 29128 |
| Zagre | 9 | 390 | 372 | 1098 | 861 | 927 | 3657 |
| Zeng | 0 | 29 | 280 | 1731 | 1110 | 624 | 3774 |
| **Total** | **452**  **(0.8%)** | **5852**  **(9.8%)** | **8979**  **(15.0%)** | **20624**  **(34.5%)** | **14064**  **(23.5%)** | **9811**  **(16.4%)** | **59782** |

**Table S5:** Multiple micronutrient supplement content and dosage by trial.

|  | **Adu-Afarwuah** | **Ashorn** | **Bhutta** | **Christian** | **Fawzi^1^** | **Friis^2^** | **Kaestel (1)^3^** | **Kaestel (2)^3^** | **MINIMat** | **Osrin** | **Roberfroid** | **West** | **Zagre** | **Zeng** |
| --- | --- | --- | --- | --- | --- | --- | --- | --- | --- | --- | --- | --- | --- | --- |
| **Iron** | 20 mg | 20 mg | 30 mg | 60 mg |  |  | 30 mg | 30 mg | 30 mg | 30 mg | 30 mg | 27 mg | 30 mg | 30 mg |
| **Folic acid** | 400 μg | 400 μg | 400 μg | 400 μg | 0.8 mg |  | 400 μg | 800 μg | 400 μg | 400 μg | 400 μg | 600 μg | 400 μg | 0.4 mg |
| **Vit A** | 800 μg | 800 μg | 800 μg | 1000 μg? |  | 3000 μg | 800 μg | 1600 μg | 800 μg | 800 μg | 800 μg | 770 μg | 800 μg | 0.8 mg |
| **Beta-carotene** |  |  |  |  |  | 3.5 mg |  |  |  |  |  |  |  |  |
| **Vit C** | 100 mg | 100 mg | 70 mg | 100 mg | 500 mg | 80 mg | 70 mg | 140 mg | 70 mg | 70 mg | 70 mg | 85 mg | 70 mg | 70 mg |
| **Vit D** | 400 IU | 400 IU | 200 IU | 10 μg |  | 10 μg | 200 IU | 400 IU | 200 IU | 5 μg | 200 IU | 5 μg | 200 IU | 5 μg |
| **Vit E** | 20 mg | 20 mg | 10 mg | 10 mg | 30 mg | 10 mg | 10 mg | 20 mg | 10 mg | 10 mg | 10 mg | 15 mg | 10 mg | 10 mg |
| **VIt B1 (thiamine)** | 2.8 mg | 2.8 mg | 1.4 mg | 1.6 mg | 20 mg | 1.5 mg | 1.4 mg | 2.8 mg | 1.4 mg | 1.4 mg | 1.4 mg | 1.4 mg | 1.4 mg | 1.4 mg |
| **Vit B2 (riboflavin)** | 2.8 mg |  | 1.4 mg | 1.8 mg | 20 mg | 1.6 mg | 1.4 mg | 2.8 mg | 1.4 mg | 1.4 mg | 1.4 mg | 1.4 mg | 1.4 mg | 1.4 mg |
| **Vit B3 (niacin)** | 36 mg | 36 mg | 18 mg | 20 mg | 100 mg | 17 mg | 18 mg | 36 mg | 18 mg | 18 mg | 18 mg | 18 mg | 18 mg | 18 mg |
| **Vit B5 (pantothenic acid)** | 7 mg | 7 mg |  |  |  |  |  |  |  |  |  |  |  |  |
| **Vit B6** | 3.8 mg | 3.8 mg | 1.9 mg | 2.2 mg | 25 mg | 2.2 mg | 1.9 mg | 3.8 mg | 1.9 mg | 1.9 mg | 1.9 mg | 1.9 mg | 1.9 mg | 1.9 mg |
| **Vit B12** | 5.2 μg | 5.2 μg | 2.6 μg | 2.6 μg | 50 μg | 4 μg | 2.6 μg | 5.2 μg | 2.6 μg | 2.6 μg | 2.6 μg | 2.6 μg | 2.6 μg | 0.026 mg |
| **Vit K** | 45 μg | 45 μg |  | 65 μg |  |  |  |  |  |  |  |  |  |  |
| **Copper** | 4 mg | 4 mg | 2 mg | 2 mg |  | 1.2 μg | 2 mg | 4 mg | 2 mg | 2 mg | 2 mg | 1000 μg | 2 mg | 2 mg |
| **Selenium** | 130 μg | 130 μg | 65 μg |  |  | 65 μg | 65 μg | 130 μg | 65 μg | 65 μg | 65 μg | 60 μg | 65 μg | 0.65 mg |
| **Zinc** | 30 mg | 30 mg | 15 mg | 30 mg |  | 15 mg | 15 mg | 30 mg | 15 mg | 15 mg | 15 mg | 12 mg | 15 mg | 15 mg |
| **Iodine** | 250 μg | 250 μg | 150 μg |  |  |  | 150 μg | 300 μg | 150 μg | 150 μg | 150 μg | 220 μg | 150 μg | 0.15 mg |
| **Magnesium** |  |  |  | 100 mg |  |  |  |  |  |  |  |  |  |  |
| **Manganese** | 2.6 mg | 2.6 mg |  |  |  |  |  |  |  |  |  |  |  |  |

^1^All women irrespective of group received daily iron (60 mg) and folic acid (0.25 mg).

^2^All women irrespective of group received daily iron and folic acid (doses not specified in paper).

^3^The Kaestel trial included two separate MMN arms; supplement (1) was used in the IPD analysis.

**Table S6:** The effect of maternal multiple micronutrient supplementation on a) low birthweight, b) preterm birth, c) stillbirth, d) neonatal death, e) perinatal death, f) maternal anemia when stratified by maternal age (<20 versus ≥20 years) and using a 2-stage IPD approach with SUMMIT trial data included.

|  | **Low birthweight** (RE)  (<2500 g) | | | **Preterm birth** (RE)  (<37 weeks) | | | **SGA** (RE)  (<10^th^ centile) | | |
| --- | --- | --- | --- | --- | --- | --- | --- | --- | --- |
|  | N (trials) | OR (95% CI) | Test for interaction | N (trials) | OR (95% CI) | Test for interaction | N (trials) | OR (95% CI) | Test for interaction |
| Overall, random effects | 14 | **0.85 (0.79-0.92)** |  | 14 | **0.88 (0.84-0.92)** |  | 14 | 0.93 (0.88-1.00) |  |
| Maternal age |  |  | P=0.84 |  |  | P=0.36 |  |  | **P=0.08** |
| <20 years |  | **0.84 (0.75-0.96)** |  |  | **0.86 (0.79-0.93)** |  |  | **0.87 (0.80-0.94)** |  |
| ≥20 years |  | **0.86 (0.77-0.95)** |  |  | **0.90 (0.84-0.97)** |  |  | 0.96 (0.88-1.05) |  |
|  | **Stillbirth** (RE)  (≥28 weeks gestation) | | | **Neonatal mortality** (RE)  (≤28 days) | | |  | | |
|  | N (trials) | OR (95% CI) | Test for interaction | N (trials) | OR (95% CI) | Test for interaction |  |  |  |
| Overall, fixed effects | 14 | 0.96 (0.86-1.08) |  | 11 | 0.99 (0.90-1.08) |  |  |  |  |
| Maternal age |  |  | P=0.34 |  |  | P=0.73 |  |  |  |
| <20 years | 11 | 1.08 (0.83-1.40) |  |  | 0.98 (0.84-1.15) |  |  |  |  |
| ≥20 years | 14 | 0.93 (0.82-1.07) |  |  | 1.02 (0.87-1.20) |  |  |  |  |

**Table S7:** The effect of maternal multiple micronutrient supplementation plus LNS compared to iron-folic acid alone on a) low birthweight, b) preterm birth, c) stillbirth, d) neonatal death, e) perinatal death, f) maternal anemia when stratified by maternal age (<20 versus ≥20 years).

|  | **Low birthweight** (RE)  (<2500 g) | | | **Preterm birth** (RE)  (<37 weeks) | | | **SGA** (RE)  (<10^th^ centile) | | |
| --- | --- | --- | --- | --- | --- | --- | --- | --- | --- |
|  | N (trials) | OR (95% CI) | Test for interaction | N (trials) | OR (95% CI) | Test for interaction | N (trials) | OR (95% CI) | Test for interaction |
| Overall, random effects | 14 | **0.86 (0.80-0.93)** |  | 14 | **0.89 (0.83-0.95)** |  | 14 | 0.93 (0.87-1.00) |  |
| Maternal age |  |  | P=0.95 |  |  | P=0.89 |  |  | **P=0.07** |
| <20 years |  | **0.86 (0.77-0.95)** |  |  | **0.89 (0.80-0.98)** |  |  | **0.88 (0.79-0.97)** |  |
| ≥20 years |  | **0.86 (0.80-0.93)** |  |  | **0.89 (0.83-0.96)** |  |  | 0.95 (0.89-1.03) |  |
|  | **Stillbirth** (FE)  (≥28 weeks gestation) | | | **Neonatal mortality** (FE)  (≤28 days) | | | **Perinatal mortality** (FE)  (≥28 weeks gestation up to ≤7 days) | | |
|  | N (trials) | OR (95% CI) | Test for interaction | N (trials) | OR (95% CI) | Test for interaction | N (trials) | OR (95% CI) | p-value |
| Overall, fixed effects | 14 | 0.98 (0.89-1.07) |  | 11 | 1.02 (0.92-1.12) |  | 12 | 0.99 (0.92-1.06) |  |
| Maternal age |  |  | **P=0.08** |  |  | P=0.62 |  |  | P=0.22 |
| <20 years |  | 1.10 (0.94-1.29) |  |  | 0.98 (0.84-1.15) |  |  | 1.06 (0.94-1.19) |  |
| ≥20 years |  | 0.93 (0.84-1.03) |  |  | 1.03 (0.92-1.17) |  |  | 0.96 (0.88-1.05) |  |
|  | **Maternal anemia (RE)**  (3^rd^ trimester Hb <110 g/L) | | |  | | | | | |
|  | N (trials) | OR (95% CI) | Test for interaction |  |  |  |  |  |  |
| Overall, random effects | 10 | **1.10 (1.00-1.21)** |  |  |  |  |  |  |  |
| Maternal age |  |  | P=0.80 |  |  |  |  |  |  |
| <20 years |  | 1.12 (0.93-1.36) |  |  |  |  |  |  |  |
| ≥20 years |  | 1.09 (0.99-1.22) |  |  | | | | | |

**Table S8a:** The effect of maternal multiple micronutrient supplementation containing iron and folic acid compared with iron-folic acid alone on birthweight (grams) when stratified by maternal age (<20 versus ≥20 years) and with removal of the West trial (Bangladesh). Two-stage IPD results presented.

| **OUTCOME** |  | **TWO-STAGE IPD** | | |
| --- | --- | --- | --- | --- |
|  |  | N (trials) | Effect size  (95% CI) | Test for Interaction |
| Birthweight (grams) | All women | 13 | +36 grams (19-54) |  |
|  | <20 years |  | +46 grams (17-75) | P=0.60 |
|  | ≥20 years |  | +34 grams (10-57) |  |

**Table S8b:** The effect of maternal multiple micronutrient supplementation containing iron and folic acid compared with iron-folic acid alone on low birthweight when stratified by maternal age (<20 versus ≥20 years) and with removal of the West trial (Bangladesh). Two-stage IPD results presented.

| **OUTCOME** |  | **TWO-STAGE IPD** | | |
| --- | --- | --- | --- | --- |
|  |  | N (trials) | OR  (95% CI) | Test for Interaction |
| Low birthweight (<2500 g) | All women | 13 | 0.89 (0.81-0.99) |  |
|  | <20 years |  | 0.86 (0.72-1.03) | P=0.65 |
|  | ≥20 years |  | 0.90 (0.80-1.03) |  |

**Table S9:** The effect of maternal multiple micronutrient supplementation containing iron and folic acid compared with iron-folic acid alone on small-for-gestational-age when stratified by maternal age (<20 versus ≥20 years) and with removal of the West trial (Bangladesh). Two-stage IPD results presented.

| **OUTCOME** |  | **TWO-STAGE IPD** | | |
| --- | --- | --- | --- | --- |
|  |  | N (trials) | OR  (95% CI) | Test for Interaction |
| SGA (<10^th^ centile) | All women | 13 | 0.95  (0.86-1.04) |  |
|  | <20 years |  | 0.86  (0.74-1.00) | P=0.36 |
|  | ≥20 years |  | 0.97  (0.86-1.11) |  |

**Table S10:** The effect of maternal multiple micronutrient supplementation containing iron and folic acid compared with iron-folic acid alone on maternal anemia when stratified by maternal age (<20 versus ≥20 years) and with removal of the West trial (Bangladesh). Two-stage IPD results presented.

| **OUTCOME** |  | **TWO-STAGE IPD** | | |
| --- | --- | --- | --- | --- |
|  |  | N (trials) | OR  (95% CI) | Test for Interaction |
| Maternal anemia (3^rd^ trimester Hb <110 g/L) | All women | 7 | 1.08 (0.93-1.24) |  |
|  | <20 years |  | 1.13 (0.87-1.47) | P=0.94 |
|  | ≥20 years |  | 1.08 (0.88-1.31) |  |

**Appendix S2: Search Strategy**

**Medline Search**

The search was executed on Medline (1946 to September Week 3, 2016) and Medline In-Process & Other Non-Indexed Citations (September 28, 2016) using the Ovid interface.

1. exp Vitamin A/ or vitamin A.tw. or exp beta Carotene/ or beta carotene*.tw. or provitamin A.tw. or exp Iodine/ or iod*.tw. or exp vitamin E/ or vitamin E.tw. or exp Ascorbic Acid/ or vitamin C.tw. or exp vitamin B 6/ or vitamin B6.tw. or exp Pyridoxine/ or pyridoxine*.tw. or exp Vitamins/ or vitamin*.tw. or exp Micronutrients/ or micronutrient*.tw. or macronutrient*.tw.
2. exp Calcium/ or calcium.tw. or exp 25-Hydroxyvitamin D 2/ or exp 25-hydroxyvitamin D 3/ or 25-Hydroxyvitamin D.tw. or exp Vitamin D/ or vitamin D.tw. or exp Zinc/ or zinc.tw. or exp Zinc Compounds/ or exp Iron/ or iron.tw. or ferr*.tw. or exp Ferric Compounds/ or exp Ferrous Compounds/ or exp Folic Acid/ or folic acid*.tw. or folate*.tw.
3. (lipid based nutrient* or lipid-based nutrient* or LNS or ready-to-use therapeutic food* or RUTF or balanced protein energy or balanced protein-energy or balanced energy protein or high protein or isocaloric protein).tw. or exp Fatty Acids, Omega-3/ or omega 3 fatty acid*.tw. or omega-3 fatty acid*.tw.
4. multiple micronutrient*.tw. or multiple macronutrient*.tw. or UNIMMAP.tw. or multimicronutrient*.tw. or multinutrient*.tw. or multi-micronutrient*.tw.
5. exp Food Habits/ or exp Nutrition Therapy/ or exp Diet Therapy/ or nutrition*.tw. or exp Food, fortified/ or fortif*.tw. or biofortif*.tw. or enrich*.tw. or exp Dietary Supplements/ or supplement*.tw. or powder*.tw. or complementary food*.tw.
6. exp Female/ or female*.tw. or exp Women/ or woman*.tw. or women.tw. or girl*.tw. or adolescen*.tw. or youth*.tw.
7. Exp Randomized Controlled Trial/ or exp Randomized Controlled Trials as Topic/ or exp Controlled Clinical Trial/ or exp Non-Randomized Controlled Trials as Topic/ or trial*.tw. or RCT*.tw. or quasi-randomized trial.tw. or quasi randomized trial.tw. or quasi experimental.tw. or quasi RCT*.tw. or exp clinical trial/ or intervention study.tw. or cluster randomiz*.tw. or randomi*.tw.
8. Systematic review*.tw. or systematic literature review.ti. or meta-analysis.pt. or meta-analysis.ti. or meta synthesis.ti. or meta-analy*.tw. or exp meta-analysis as topic/
9. (1 or 2 or 3 or 4) and 5 and 6 and 7 and 8
10. Limit 9 to English

**Appendix S3: Risk of Bias Assessment**

**Adu-Afarwuah^S1^:**

| **Bias** | **Authors’ Judgement** | **Support for Judgement** |
| --- | --- | --- |
| Random sequence generation (selection bias) | Low risk | Quote: "women were randomly allocated into one of 3 groups by using a computer-generated scheme (SAS version 9.3; SAS Institute) in blocks of 9."  Comment: probably done |
| Allocation concealment (selection bias) | Low risk | Quote: "Sheets bearing supplement allocations represented by 6 different color codes (3 for IFA and 3 for MMN) and an inscription “LNS” (for the LNS group) and numbered 1–1320 were placed in opaque envelopes and stacked in increasing order. At each enrolment, the study nurse shuffled the 9 topmost envelopes in the stack, and the woman picked one to reveal allocation. Allocation information was kept by the field supervisor (HO) in a password-protected file, which was shared with the study statistician (JMP) at UC Davis, who designed the randomisation scheme."  Comment: probably done |
| Blinding of participants and personnel (performance bias) | Low risk | Quote: "Two individuals in Ghana who were independent of the research team color-coded the capsules by placing color stickers (which also included the letter P or L to indicate pregnancy or lactation) on the blister packs of IFA and MMN, so that no investigator, study worker, or participant knew the identities of the capsules except by the colors."  Comment: participants and caregivers were probably blinded to the treatment assignment. |
| Blinding of outcome assessment (detection bias) | Low risk | Quote: "none of the maternal or newborn anthropometrists was aware of the code allocations. Likewise, data analysts remained blinded until all preliminary analyses had been completed, and the allocation codes were broken."  Comment: outcome assessors were probably blinded to the treatment assignment. |
| Incomplete outcome data (attrition bias) | Low risk | Exclusion (until delivery) was 20% (and was balanced between treatment arms); the reason was reported. Attrition (until delivery) was 4.4% and reasons were reported |
| Selective reporting (reporting bias) | Low risk | Comment: results of all outcomes mentioned in methods section were presented in the paper |
| Other bias | Low risk | Comment: no other bias was identified |

**Ashorn^S2^:**

| **Bias** | **Authors’ Judgement** | **Support for Judgement** |
| --- | --- | --- |
| Random sequence generation (selection bias) | Low risk | Quote: "A study statistician not involved in data collection generated 4 randomization code lists in blocks of 9 (one list for each of the 4 enrolment sites)."  Comment: probably done |
| Allocation concealment (selection bias) | Low risk | Quote: "a researcher not involved with the trial created individual randomisation slips (in blocks of 9) and packed them in sealed, numbered, opaque randomisation envelopes that were stored in numerical order. Eligible pregnant women were requested to choose 1 of the top 6 envelopes in the stack, and the contents of the envelope indicated her participant number and group allocation."  Comment: probably done |
| Blinding of participants and personnel (performance bias) | Low risk | Quote: "The IFA and MMN interventions were provided by using double-masked procedures—that is, the capsules looked identical, and neither the participants nor the research team members were aware of the nutrient contents of the supplement capsules."  Comment: participants and caregivers were probably blinded to the treatment assignment. |
| Blinding of outcome assessment (detection bias) | Low risk | Quote: "The data collectors who performed the anthropometric measurements or assessed other outcomes were not aware of group allocation. Researchers responsible for the data cleaning remained blind to the trial code until the database was fully cleaned."  Comment: outcome assessors were probably blinded to the treatment assignment. |
| Incomplete outcome data (attrition bias) | Low risk | Attrition (until delivery) was 6.0% (and was balanced between treatment arms); reasons were not reported |
| Selective reporting (reporting bias) | Low risk | Comment: all outcomes presented in the methods section were reported in the paper. |
| Other bias | Low risk | Comment: no other bias was identified. |

**Bhutta^S3^:**

| **Bias** | **Authors’ Judgement** | **Support for Judgement** |
| --- | --- | --- |
| Random sequence generation (selection bias) | Low risk | Quote: "a cluster-based allocation strategy of supplements (either IF or MMN supplementation) by respective CHWs was implemented".  Comment: probably done |
| Allocation concealment (selection bias) | Low risk | Comment: "allocated to either the IF or MMN supplements according to their respective location and allocation by the AKU Pharmacy".  Comment: probably done |
| Blinding of participants and personnel (performance bias) | Low risk | Quote: "Both tablets were identical in colour, shape and packaging" and "field staff (medical officers, CHWs, social scientists and data collection team) remained completely blinded as to the supplements allocation. All pregnant women were allocated a unique code and allocated a uniquely labelled and numerically coded specific supplement supply". Comment: participants and caregivers were probably blinded to the treatment assignment. |
| Blinding of outcome assessment (detection bias) | Low risk | Quote: "Both tablets were identical in colour, shape and packaging" and "field staff (medical officers, CHWs, social scientists and data collection team) remained completely blinded as to the supplements allocation".  Comment: outcome assessors were probably blinded to the treatment assignment. |
| Incomplete outcome data (attrition bias) | Low risk | Attrition (15.8%) and exclusion (around 1%) along with their reasons were reported. Attrition and exclusions were balanced across the treatment arms. |
| Selective reporting (reporting bias) | Low risk | Comment: results of all outcomes mentioned in methods section were presented in the paper |
| Other bias | Low risk | Comment: no other bias was identified, including cluster-design specific biases (recruitment bias, baseline imbalance, loss of clusters, incorrect analysis, and comparability with individually randomised trials) |

**Christian^S4^:**

| **Bias** | **Authors’ Judgement** | **Support for Judgement** |
| --- | --- | --- |
| Random sequence generation (selection bias) | Low risk | Quote: "Randomisation was done in blocks of five within each village development community by the senior study investigators, who drew numbered chips from a hat"  Comment: probably done |
| Allocation concealment (selection bias) | Unclear risk | Quote: "Randomisation was done in blocks of five within each village development community by the senior study investigators, who drew numbered chips from a hat"  Comment: insufficient information to permit judgement |
| Blinding of participants and personnel (performance bias) | Low risk | Quote: "participants, investigators, field staff and statisticians did not know supplement codes", "supplements, which were of identical shape, size, and color" and "code allocation was kept locked at the Johns Hopkins University, Baltimore". Comment: participants and caregivers were blinded to the treatment assignment. |
| Blinding of outcome assessment (detection bias) | Low risk | Quote: "participants, investigators, field staff and statisticians did not know supplement codes" Comment: outcome assessors were blinded to the treatment assignment. |
| Incomplete outcome data (attrition bias) | Low risk | Exclusion (1.43%) and attrition (6.9%) were reported along with their reasons |
| Selective reporting (reporting bias) | Low risk | Comment: results of all outcomes mentioned in methods were presented in the various publications of this trial. |
| Other bias | Low risk | Comment: no other bias was identified, including cluster-design specific biases (recruitment bias, baseline imbalance, loss of clusters, incorrect analysis, and comparability with individually randomised trials). |

**Fawzi^S5^:**

| **Bias** | **Authors’ Judgement** | **Support for Judgement** |
| --- | --- | --- |
| Random sequence generation (selection bias) | Low risk | Quote: "A list was prepared according to a randomisation sequence in blocks of 20; at enrolment, each eligible women was assigned to the next numbered bottle" and computerised random number generator was used (personal communication)  Comment: probably done |
| Allocation concealment (selection bias) | Low risk | Quote: "Each eligible women was assigned to the next numbered bottle"  Comment: probably done |
| Blinding of participants and personnel (performance bias) | Low risk | Quote: "Active tablets and placebo were similar in shape, size and color and were packaged in identical coded bottles" and "Each eligible women was assigned to the next numbered bottle" Comment: participants and caregivers were blinded to the treatment assignment. |
| Blinding of outcome assessment (detection bias) | Low risk | Quote: "research assistants who assessed the study outcome were unaware of the intervention group"  Comment: outcome assessors were blinded. |
| Incomplete outcome data (attrition bias) | Low risk | Exclusion (0.5%) and attrition (5.4%) were reported with reasons in each arm. |
| Selective reporting (reporting bias) | Low risk | Comment: all outcomes mentioned in the methods section were presented in the paper. |
| Other bias | Low risk | Comment: no other bias was identified. |

**Friis^S6^:**

| **Bias** | **Authors’ Judgement** | **Support for Judgement** |
| --- | --- | --- |
| Random sequence generation (selection bias) | Low risk | Quote: "Allocation to daily supplementation with multimicronutrient or identical-looking placebo tablets was based on simple blocked randomisation. The digits 0–5 in a computer-generated random sequence were replaced by 6 preassigned permuted blocks of 4: AABB, ABAB, ABBA, BABA, BBAA, and BAAB; the digits 6–9 were deleted".  Comment: probably done |
| Allocation concealment (selection bias) | Low risk | Quote: "Containers with 110 multimicronutrient or placebo tablets, which were coded A or B, respectively, were delivered by the manufacturer together with the code in 2 sealed envelopes. Duplicate containers, which corresponded to the random sequence, were consecutively numbered from 1 to 1800. The study participants were numbered consecutively at recruitment".  Comment: probably done |
| Blinding of participants and personnel (performance bias) | Low risk | Quote: "double blind", "multimicronutrient or identical-looking placebo tablets" Comment: study participants and care providers were probably blinded to the treatment assignment. |
| Blinding of outcome assessment (detection bias) | Low risk | Quote: "double blind", "multimicronutrient or identical-looking placebo tablets"  Comment: investigators were probably blinded to the treatment assignment. |
| Incomplete outcome data (attrition bias) | High risk | Attrition was > 20% and reasons for it were reported. Exclusions were not reported in the trial |
| Selective reporting (reporting bias) | Low risk | Comment: all outcomes in the methods section were presented in the paper. |
| Other bias | Low risk | Comment: no other bias was identified. |

**Kaestel^S7^:**

| **Bias** | **Authors’ Judgement** | **Support for Judgement** |
| --- | --- | --- |
| Random sequence generation (selection bias) | Low risk | Quote: "Simple block randomisation with a block size of 150 was managed as follows: at entry, the project midwife randomly drew 1 piece of coloured paper corresponding to the colour code on the tablet containers from envelopes with initially 50 pieces of each of the three colours"  Comment: probably done. |
| Allocation concealment (selection bias) | Unclear risk | Quote: "at entry, the project midwife randomly drew one piece of coloured paper corresponding to the colour code on the tablet containers from envelopes with initially 50 pieces of each of the three colours"  Comment: insufficient evidence to determine whether allocation was concealed following generation of the randomisation sequence. |
| Blinding of participants and personnel (performance bias) | Low risk | Quote: "three identical-looking micronutrient supplements", "code was kept secret from study participants, study personnel, and data analysts until data cleaning and preliminary data analysis had been carried out." and "the health workers who collected outcome data after delivery did not have any knowledge of intervention group of the women" Comment: participants and caregivers were probably blinded to the treatment assignment. |
| Blinding of outcome assessment (detection bias) | Low risk | Quote: "three identical-looking micronutrient supplements", "code was kept secret from study participants, study personnel, and data analysts until data cleaning and preliminary data analysis had been carried out." and "the health workers who collected outcome data after delivery did not have any knowledge of intervention group of the women" Comment: outcome assessors were probably blinded to the treatment assignment. |
| Incomplete outcome data (attrition bias) | High risk | Exclusion (3.1%) and attrition (20.4%) data were reported along with their reasons. |
| Selective reporting (reporting bias) | Low risk | Comment: all outcomes mentioned in the methods section were presented in the paper. |
| Other bias | Low risk | Comment: no other bias was identified. |

**MINIMAt^S8^:**

| **Bias** | **Authors’ Judgement** | **Support for Judgement** |
| --- | --- | --- |
| Random sequence generation (selection bias) | Unclear risk | Quote: "individual randomisation was done in blocks of 12" and "After enrolment, women were randomly assigned to 6 intervention groups".  Comment: method used for generating the randomisation sequence was not described in sufficient detail to permit judgement. |
| Allocation concealment (selection bias) | Unclear risk | Comment: method used for allocation concealment was not described in sufficient detail to permit judgement. |
| Blinding of participants and personnel (performance bias) | Low risk | Quote: "pills were identical in appearance, and monthly supplies were provided in identical bottles", " the mothers were unaware of their micronutrient supplement" and "double masking was practiced" Comment: study participants and caregivers were blinded to the treatment assignment. |
| Blinding of outcome assessment (detection bias) | Low risk | Quote: "pills were identical in appearance, and monthly supplies were provided in identical bottles", "the testers were unaware of children’s groups" and "double masking was practiced" Comment: outcome assessors were blinded to the treatment assignment. |
| Incomplete outcome data (attrition bias) | High risk | Attrition was (26%), reported along with their reasons. |
| Selective reporting (reporting bias) | Low risk | Comment: all outcomes mentioned in the methods section were presented in the paper. |
| Other bias | Low risk | Comment: no other bias was identified. |

**Osrin^S9^:**

| **Bias** | **Authors’ Judgement** | **Support for Judgement** |
| --- | --- | --- |
| Random sequence generation (selection bias) | Low risk | Quote: "Randomly allocated 1200 participant identification numbers by computer into two groups in permuted blocks of 50".  Comment: probably done |
| Allocation concealment (selection bias) | Low risk | Quote: "We did randomisation in advance of recruitment", "The allocation code was kept on file in Kathmandu and London. We allocated every identification number a supplement container to last throughout the trial. Containers were filled with either intervention or control tablets in Kathmandu by a team member who was otherwise uninvolved in the trial; these containers were then marked only with identification numbers and transported to the study centre in Janakpur" and "After screening, consent, and enrolment, one of us (YS) allocated participants sequential identification numbers and the corresponding supplement containers".  Comment: probably done |
| Blinding of participants and personnel (performance bias) | Low risk | Quote: "The allocation code was kept on file in Kathmandu and London" and "Containers were filled with either intervention or control tablets in Kathmandu by a team member who was otherwise uninvolved in the trial; these containers were then marked only with identification numbers and transported to the study centre in Janakpur. Intervention and control supplements were manufactured to look, smell, and taste identical" Comment: participants and caregivers were probably blinded to the treatment assignment. |
| Blinding of outcome assessment (detection bias) | Low risk | Quote: "The allocation code was kept on file in Kathmandu and London" and "Containers were filled with either intervention or control tablets in Kathmandu by a team member who was otherwise uninvolved in the trial; these containers were then marked only with identification numbers and transported to the study centre in Janakpur. Intervention and control supplements were manufactured to look, smell, and taste identical" Comment: outcome assessors were probably blinded to the treatment assignment. |
| Incomplete outcome data (attrition bias) | High risk | Attrition was 5% and reasons for it were reported. Exclusion was 39.5% and reasons were not reported. |
| Selective reporting (reporting bias) | Low risk | Comment: all outcomes mentioned in the methods section were presented in the paper. |
| Other bias | Low risk | Comment: no other bias was identified. |

**Roberfroid^S10^:**

| **Bias** | **Authors’ Judgement** | **Support for Judgement** |
| --- | --- | --- |
| Random sequence generation (selection bias) | Low risk | Quote: "The randomisation scheme was generated by a computer program in permuted blocks of 4".  Comment: probably done |
| Allocation concealment (selection bias) | Low risk | Quote: "Randomization numbers were sealed in opaque envelopes. At each inclusion, the consulting physician opened the next sealed envelope and transmitted the randomisation number to a pharmacist managing the allocation sequence and the packaging of drugs in Center Muraz. The pharmacist was also blinded to the intervention. Individual plastic zip bags contained 31 tablets each and were labelled with the participant’s name, address, and identification numbers only"  Comment: probably done |
| Blinding of participants and personnel (performance bias) | Low risk | Quote: "double blind", "Intervention and control micronutrient tablets were identical in appearance" and "code was kept secret from study participants and staff until completion of preliminary data analysis" and "Pharmacist was also blinded to the intervention". Comment: participants and caregivers were probably blinded to the treatment assignment. |
| Blinding of outcome assessment (detection bias) | Low risk | Quote: "double blind", "Intervention and control micronutrient tablets were identical in appearance" and "code was kept secret from study participants and staff until completion of preliminary data analysis" and "Pharmacist was also blinded to the intervention". Comment: outcome assessors were probably blinded to the treatment assignment. |
| Incomplete outcome data (attrition bias) | Low risk | Attrition was 7.5% and reason for it was provided. Only 1 woman was excluded because of therapeutic abortion. |
| Selective reporting (reporting bias) | Low risk | Comment: all outcomes mentioned in the methods section were presented in the paper. |
| Other bias | Low risk | Comment: no other bias was identified. |

**West^S11^:**

| **Bias** | **Authors’ Judgement** | **Support for Judgement** |
| --- | --- | --- |
| Random sequence generation (selection bias) | Low risk | Quote: "We used an in-house program (VBScript, Microsoft) that recognized 70 possible permutations for n=8 sectors and k=2 supplement allocations and 6 for the last block of n=4 sectors. Using this program, we randomized sectors within blocks to 1 of 2 codes such that each permutation had an equal probability of being chosen." |
| Allocation concealment (selection bias) | Low risk | Quote: "The resulting 2 lists of sectors were securely transmitted to field headquarters. One envelope with the code key was securely transmitted to the supplement producer and the other sealed in an envelope and secured at Johns Hopkins. At no time during the trial did study investigators or field or data management staff have access to the key." |
| Blinding of participants and personnel (performance bias) | Low risk | Quote: "double-masked", "Double Blind (Subject, Caregiver, Investigator, Outcomes Assessor)", "received daily supplementation, so treatment effect (still blinded due to the ongoing trial)"  Comment: probably done |
| Blinding of outcome assessment (detection bias) | Low risk | Quote: "double-masked", "Double Blind (Subject, Caregiver, Investigator, Outcomes Assessor)", "received daily supplementation, so treatment effect (still blinded due to the ongoing trial)"  Comment: probably done |
| Incomplete outcome data (attrition bias) | Low risk | Complete information was not available as the main trial has not been published; however, attrition is reported to be < 20% (trial presentations). |
| Selective reporting (reporting bias) | Low risk | Comment: reports from the study are still being published |
| Other bias | Low risk | Comment: no other bias was identified, including cluster-design specific biases (recruitment bias, baseline imbalance, loss of clusters, incorrect analysis, and comparability with individually randomised trials) |

**Zagre^S12^:**

| **Bias** | **Authors’ Judgement** | **Support for Judgement** |
| --- | --- | --- |
| Random sequence generation (selection bias) | Unclear risk | Quote: "Villages - not individuals were randomly assigned to one treatment group or the other"  Comment: method used for generating the randomisation sequence was not described in sufficient detail to permit judgement. |
| Allocation concealment (selection bias) | Unclear risk | Comment: method used for allocation concealment was not described to permit judgement. |
| Blinding of participants and personnel (performance bias) | Low risk | Quote: "Because the two supplements did not look identical and may have been recognizable, a coding system was put in place by the SONIPHAR pharmaceutical company in Niger. Six codes were assigned to the treatments: three for iron/folic acid and three for multimicronutrient supplements. SONIPHAR packaged the supplements in boxes with identical labelling except for the supplement code. Health workers, traditional midwives, and data collectors were informed that each supplement came in two sizes and colors, so that the code letter did not distinguish which supplement was used". Comment: participants and caregivers were probably blinded to the treatment assignment. |
| Blinding of outcome assessment (detection bias) | Low risk | Quote: "Because the two supplements did not look identical and may have been recognizable, a coding system was put in place by the SONIPHAR pharmaceutical company in Niger. six codes were assigned to the treatments: three for iron/folic acid and three for multimicronutrient supplements. SONIPHAR packaged the supplements in boxes with identical labelling except for the supplement code. Health workers, traditional midwives, and data collectors were informed that each supplement came in two sizes and colors, so that the code letter did not distinguish which supplement was used". Comment: outcome assessors were probably blinded to the treatment assignment. |
| Incomplete outcome data (attrition bias) | Unclear risk | Attrition was 18%. Reasons for attrition were reported, and dropout was significantly higher in the MMN (25/1893 (1.3%)) versus IFA (8/1777 (0.5%)) group. Exclusion data were not reported. |
| Selective reporting (reporting bias) | Low risk | Comment: all outcomes mentioned in the methods section were presented in the paper. |
| Other bias | Low risk | Comment: no other bias was identified, including cluster-design-specific biases (recruitment bias, baseline imbalance, loss of clusters, and comparability with individually randomised trials). Any incorrect analysis was corrected by adjustment for clustering within data reported in this review. |

**Zeng^S13^:**

| **Bias** | **Authors’ Judgement** | **Support for Judgement** |
| --- | --- | --- |
| Random sequence generation (selection bias) | Low risk | Quote: "The randomisation schedule was generated off site with a pseudo-random number generator in SAS".  Comment: probably done |
| Allocation concealment (selection bias) | Low risk | Quote: "The randomisation schedule was generated off site with a pseudo-random number generator in SAS version 6 (SAS Institute, Cary, NC). A treatment colour code was assigned to each village based on the treatment allocation schedule".  Comment: probably done |
| Blinding of participants and personnel (performance bias) | Low risk | Quote: "double blind", "treatment colour code was assigned to each village based on the treatment allocation schedule. The treatment codes were opened only once all data had been collected and blinded analysis of the primary hypothesis was completed" and "were of identical appearance and packaged in blister packs"  Comment: participants and caregivers were blinded to the treatment assignment. |
| Blinding of outcome assessment (detection bias) | Low risk | Quote: "double blind", "treatment colour code was assigned to each village based on the treatment allocation schedule. The treatment codes were opened only once all data had been collected and blinded analysis of the primary hypothesis was completed"  Comment: outcome assessors were blinded to the treatment assignment. |
| Incomplete outcome data (attrition bias) | Low risk | Exclusion (4.8%) and attrition (2.3%) were reported along with their reasons |
| Selective reporting (reporting bias) | Low risk | Comment: all outcomes mentioned in the methods section were presented in the paper |
| Other bias | Low risk | Comment: no other bias was identified, including cluster-design-specific biases (recruitment bias, baseline imbalance, loss of clusters, and comparability with individually randomised trials). Investigators did not adjust for the cluster-randomised design in their sample size or outcome estimations, but this was corrected. |

References:

S1) Adu-Afarwuah S, Lartey A, Okronipa H, et al. Lipid-based nutrient supplement increases the birth size of infants of primiparous women in ghana. *Am J Clin Nutr.* 2015;101(4):835-846. doi: 10.3945/ajcn.114.091546.

S2) Ashorn P, Alho L, Ashorn U, et al. The impact of lipid-based nutrient supplement provision to pregnant women on newborn size in rural malawi: A randomized controlled trial. *Am J Clin Nutr.* 2015;101(2):387-397. doi: 10.3945/ajcn.114.088617.

S3) Bhutta ZA, Rizvi A, Raza F, et al. A comparative evaluation of multiple micronutrient and iron-folic acid supplementation during pregnancy in pakistan: Impact on pregnancy outcomes. *Food Nutr Bull.* 2009;30(4 Suppl):S496-505. doi: 10.1177/15648265090304S404.

S4) Christian P, Khatry SK, Katz J, et al. Effects of alternative maternal micronutrient supplements on low birth weight in rural nepal: Double blind randomised community trial. *BMJ.* 2003;326(7389):571. doi: 10.1136/bmj.326.7389.571.

S5) Fawzi WW, Msamanga GI, Urassa W, et al. Vitamins and perinatal outcomes among hiv-negative women in tanzania. *N Engl J Med.* 2007;356(14):1423-1431. doi: 10.1056/NEJMoa064868.

S6) Friis H, Gomo E, Nyazema N, et al. Effect of multimicronutrient supplementation on gestational length and birth size: A randomized, placebo-controlled, double-blind effectiveness trial in zimbabwe. *Am J Clin Nutr.* 2004;80(1):178-184. doi: 10.1093/ajcn/80.1.178.

S7) Kaestel P, Michaelsen KF, Aaby P, Friis H. Effects of prenatal multimicronutrient supplements on birth weight and perinatal mortality: A randomised, controlled trial in guinea-bissau. *Eur J Clin Nutr.* 2005;59(9):1081-1089. doi: 10.1038/sj.ejcn.1602215.

S8) Persson LA, Arifeen S, Ekstrom EC, et al. Effects of prenatal micronutrient and early food supplementation on maternal hemoglobin, birth weight, and infant mortality among children in bangladesh: The minimat randomized trial. *JAMA.* 2012;307(19):2050-2059. doi: 10.1001/jama.2012.4061.

S9) Osrin D, Vaidya A, Shrestha Y, et al. Effects of antenatal multiple micronutrient supplementation on birthweight and gestational duration in nepal: Double-blind, randomised controlled trial. *Lancet.* 2005;365(9463):955-962. doi: 10.1016/S0140-6736(05)71084-9.

S10) Roberfroid D, Huybregts L, Lanou H, et al. Effects of maternal multiple micronutrient supplementation on fetal growth: A double-blind randomized controlled trial in rural burkina faso. *Am J Clin Nutr.* 2008;88(5):1330-1340. doi: 10.3945/ajcn.2008.26296.

S11) West KP, Jr., Shamim AA, Mehra S, et al. Effect of maternal multiple micronutrient vs iron-folic acid supplementation on infant mortality and adverse birth outcomes in rural bangladesh: The jivita-3 randomized trial. *JAMA.* 2014;312(24):2649-2658. doi: 10.1001/jama.2014.16819.

S12) Zagre NM, Desplats G, Adou P, Mamadoultaibou A, Aguayo VM. Prenatal multiple micronutrient supplementation has greater impact on birthweight than supplementation with iron and folic acid: A cluster-randomized, double-blind, controlled programmatic study in rural niger. *Food Nutr Bull.* 2007;28(3):317-327. doi: 10.1177/156482650702800308.

S13) Zeng L, Dibley MJ, Cheng Y, et al. Impact of micronutrient supplementation during pregnancy on birth weight, duration of gestation, and perinatal mortality in rural western china: Double blind cluster randomised controlled trial. *BMJ.* 2008;337:a2001. doi: 10.1136/bmj.a2001.

**Appendix S4: PRISMA Checklist^S1^**

| **Section/topic** | **#** | **Checklist item** | **Reported on page #** |
| --- | --- | --- | --- |
| **TITLE** | | |  |
| Title | 1 | Identify the report as a systematic review, meta-analysis, or both. | Pg 6 (systematic review of reviews); Pg 8 (IPD meta-analysis) |
| **ABSTRACT** | | |  |
| Structured summary | 2 | Provide a structured summary including, as applicable: background; objectives; data sources; study eligibility criteria, participants, and interventions; study appraisal and synthesis methods; results; limitations; conclusions and implications of key findings; systematic review registration number. | Pg 2-3 |
| **INTRODUCTION** | | |  |
| Rationale | 3 | Describe the rationale for the review in the context of what is already known. | Pg 4-5 |
| Objectives | 4 | Provide an explicit statement of questions being addressed with reference to participants, interventions, comparisons, outcomes, and study design (PICOS). | Pg 5-6 |
| **METHODS** | | |  |
| Protocol and registration | 5 | Indicate if a review protocol exists, if and where it can be accessed (e.g., Web address), and, if available, provide registration information including registration number. | Protocol not registered |
| Eligibility criteria | 6 | Specify study characteristics (e.g., PICOS, length of follow-up) and report characteristics (e.g., years considered, language, publication status) used as criteria for eligibility, giving rationale. | Pg 6-7; Table 1 |
| Information sources | 7 | Describe all information sources (e.g., databases with dates of coverage, contact with study authors to identify additional studies) in the search and date last searched. | Pg 6-7 |
| Search | 8 | Present full electronic search strategy for at least one database, including any limits used, such that it could be repeated. | Appendix II |
| Study selection | 9 | State the process for selecting studies (i.e., screening, eligibility, included in systematic review, and, if applicable, included in the meta-analysis). | Pg 6-7 |
| Data collection process | 10 | Describe method of data extraction from reports (e.g., piloted forms, independently, in duplicate) and any processes for obtaining and confirming data from investigators. | Pg 7 (we requested individual level data) |
| Data items | 11 | List and define all variables for which data were sought (e.g., PICOS, funding sources) and any assumptions and simplifications made. | Pg 7-8 (outcomes and covariates of interest) |
| Risk of bias in individual studies | 12 | Describe methods used for assessing risk of bias of individual studies (including specification of whether this was done at the study or outcome level), and how this information is to be used in any data synthesis. | Pg 7 (Appendix III) |
| Summary measures | 13 | State the principal summary measures (e.g., risk ratio, difference in means). | Pg 9-10 |
| Synthesis of results | 14 | Describe the methods of handling data and combining results of studies, if done, including measures of consistency (e.g., I^2^) for each meta-analysis. | Pg 9-11 |
| Section/topic | # | Checklist item | Reported on page # |
| Risk of bias across studies | 15 | Specify any assessment of risk of bias that may affect the cumulative evidence (e.g., publication bias, selective reporting within studies). | Pg 19 (selection bias when randomization not preserved due to stratification of adolescent subgroup) |
| Additional analyses | 16 | Describe methods of additional analyses (e.g., sensitivity or subgroup analyses, meta-regression), if done, indicating which were pre-specified. | Pg 7; Pg 11 |
| **RESULTS** | | |  |
| Study selection | 17 | Give numbers of studies screened, assessed for eligibility, and included in the review, with reasons for exclusions at each stage, ideally with a flow diagram. | Figure 1 |
| Study characteristics | 18 | For each study, present characteristics for which data were extracted (e.g., study size, PICOS, follow-up period) and provide the citations. | Table 2 (no data extraction beyond study characteristics, as we had individual level data); Appendix I, Tables 4 and 5 |
| Risk of bias within studies | 19 | Present data on risk of bias of each study and, if available, any outcome level assessment (see item 12). | Appendix III |
| Results of individual studies | 20 | For all outcomes considered (benefits or harms), present, for each study: (a) simple summary data for each intervention group (b) effect estimates and confidence intervals, ideally with a forest plot. | Figures 2 and 3 |
| Synthesis of results | 21 | Present results of each meta-analysis done, including confidence intervals and measures of consistency. | Tables 3a and 3b (main results) and 4 (effect modification) |
| Risk of bias across studies | 22 | Present results of any assessment of risk of bias across studies (see Item 15). | Pg 19 |
| Additional analysis | 23 | Give results of additional analyses, if done (e.g., sensitivity or subgroup analyses, meta-regression [see Item 16]). | Sensitivity analyses: Appendix I, Tables 6-10 |
| **DISCUSSION** | | |  |
| Summary of evidence | 24 | Summarize the main findings including the strength of evidence for each main outcome; consider their relevance to key groups (e.g., healthcare providers, users, and policy makers). | Pg 15-16; Pg 19-20 |
| Limitations | 25 | Discuss limitations at study and outcome level (e.g., risk of bias), and at review-level (e.g., incomplete retrieval of identified research, reporting bias). | Pg 16-17 |
| Conclusions | 26 | Provide a general interpretation of the results in the context of other evidence, and implications for future research. | Pg 17-19 |
| **FUNDING** | | |  |
| Funding | 27 | Describe sources of funding for the systematic review and other support (e.g., supply of data); role of funders for the systematic review. | Pg 23-24 |

References:

S1) Moher D, Liberati A, Tetzlaff J, Altman DG, The PRISMA Group (2009). Preferred Reporting Items for Systematic Reviews and Meta-Analyses: The PRISMA Statement. PLoS Med 6(7): e1000097. doi:10.1371/journal.pmed1000097.
